# Anisotropic alignments of hierarchical Li_2_SiO_3_/TiO_2_ @nano-C anode//LiMnPO_4_@nano-C cathode architectures for full-cell lithium-ion battery

**DOI:** 10.1093/nsr/nwaa017

**Published:** 2020-02-11

**Authors:** Hesham Khalifa, Sherif A El-Safty, Abdullah Reda, Mohamed A Shenashen, Alaa I Eid

**Affiliations:** 1 Research Center for Functional Materials, National Institute for Materials Science, Tsukuba 305-0047, Japan; 2 Department of Petrochemical, Egyptian Petroleum Research Institute, Cairo 11727, Egypt; 3 Composite Lab, Advanced Materials Division, Central Metallurgical R&D Institute, Helwan 11421, Egypt

**Keywords:** lithium-ion batteries, micro/nano architectures, LiMnPO_4_@nano-carbon cathode, Li_2_SiO_3_/TiO_2_@nano-carbon anode, LIB-CR2032 coin cell models

## Abstract

We report on low-cost fabrication and high-energy density of full-cell lithium-ion battery (LIB) models. Super-hierarchical electrode architectures of Li_2_SiO_3_/TiO_2_@nano-carbon anode (LSO.TO@nano-C) and high-voltage olivine LiMnPO_4_@nano-carbon cathode (LMPO@nano-C) are designed for half- and full-system LIB-CR2032 coin cell models. On the basis of primary architecture-power-driven LIB geometrics, the structure keys including three-dimensional (3D) modeling superhierarchy, multiscale micro/nano architectures and anisotropic surface heterogeneity affect the buildup design of anode/cathode LIB electrodes. Such hierarchical electrode surface topologies enable continuous in-/out-flow rates and fast transport pathways of Li^+^-ions during charge/discharge cycles. The stacked layer configurations of pouch LIB-types lead to excellent charge/discharge rate, and energy density of 237.6 Wh kg^−1^. As the most promising LIB-configurations, the high specific energy density of hierarchical pouch battery systems may improve energy storage for long-driving range of electric vehicles. Indeed, the anisotropic alignments of hierarchical electrode architectures in the large-scale LIBs provide proof of excellent capacity storage and outstanding durability and cyclability. The full-system LIB-CR2032 coin cell models maintain high specific capacity of ∼89.8% within a long-term life period of 2000 cycles, and average Coulombic efficiency of 99.8% at 1C rate for future configuration of LIB manufacturing and commercialization challenges.

## INTRODUCTION

The advancement of new anode/cathode materials with improved performance and safety currently dominates research on lithium-particle battery-powered batteries [lithium-ion batteries (LIBs)]. The design-build LIBs have shown brilliant future applications and cutting-edge versatility in terms of clean, environmentally friendly, sustainable energy, offering a power source for modern electronic devices [1,2]. Among different types of rechargeable batteries, LIBs are the most fortified candidate because of their unique features, including high power and energy density, high operating voltage, low self-discharge, long life cycle and environmentally friendly materials. LIBs are increasingly important as technology in zero-emission transportation using electric vehicles (EVs), and plug-in hybrid electric vehicles (PHEVs) [3–6]. LIBs show potential in sustainable energy because of their remarkable volumetric and gravimetric energy and power densities [3–6], providing a minimum driving mileage of ∼300 miles (energy storage of approximately 100 Wh kg^−1^). This preceding stage ensures the future of EVs in the marketplace [7]. The automobile market worldwide is facing development challenges in terms of cost to achieve this range. In this context, much effort has been devoted to design a new sustainable anode/cathode system that is cheap, safe, environmentally friendly, and has high energy density, specific capacity and voltage.

Silicon is a low-cost, safe and ecological material, and is relatively abundant (26.3% by weight) in Earth's crust. Silicon has a special niche for the design of negative (N) electrodes in LIBs because of its excellent theoretical gravimetric capacity. For instance, the N-electrode Li_15_Si_4_, and Li_22_Si_4_ structures showed capacity of 3590 and 4200 mAh g^−1^, respectively, at room temperature [8–12]. Silicon N-electrode has a capacity nine times higher than that of graphite as a negative electrode material, and has low operation voltage for electrochemical alloy/de-alloy (0.4–0.5 V versus Li/Li^+^) [13–16]. However, silicon implementation is limited given the significant loss of irreversible capacity because of fracture, particle pulverization and strong mechanical strain cracking; the electrical contact is lost between the particles, and a solid-electrolyte interface film (SEI) is constantly built up along the surface of the anode [15–20] from great volume swing (swell and retraction) of up to 300–400% during lithiation/delithiation cycling [21,22]. These phenomena result in poor electrical conductivity [23–25]. The SEI represents the passivation layer formed between solid-electrode/electrolyte, with a key role in formation of electronically insulated and ionically conductive solid electrolyte behaviors [26]. To overcome these obstacles of conductivity, scholars have focused on developing and reviewing available strategies to fill the key knowledge gaps in use of nano-scale architectures or multi-reactive centering components and the optimized performance of silicon-based composite LIB anodes [27,28]. Among Si composites, lithium metasilicate Li_2_SiO_3_ (LSO) composite can serve as an excellent anode material because of its stable structure, good conductivity and ability to provide typical Li^+^-ion in-/out-flow rates, and 3D-diffusion tunnel. Furthermore, TiO_2_-anatase (TO) is an excellent anode material, providing outstanding design-build LIB advantages with high safety performance as a result of its eco-friendliness, low cost, non-toxicity, pollution-free nature and low polarization [29–31]. The mobility that dominates its structure transition from I41/amd (space group)-tetragonal TiO_2_ to Imma (space group)-orthorhombic lithium-rich Li_0.5_TiO_2_ geometrics is a key benefit when building anodic electrodes. These N-electrode features enabled fabrication of LIB designs with good cyclic stability and reversibility [29–31]. The atomic-scale rearrangements and spontaneous phase transitions along the TO structures from poor-to-rich lithium structures (i.e. Li_0.01_TiO_2_ to Li_0.5_TiO_2_ composition geometrics) offered excellent electrode surface mobility and flexibility during multiple charge/discharge processes [32]. Therefore, integration of lithium-rich LSO and TO nanocomposites and architectures is a key factor for improving electrochemical performance of LIB geometrics.

Multiscale cathode architectures of lithium-rich composites would boost the LIB half-cell geometrics with multi-gateway migrations and unlimited mass transports of Li^+^ ions. Among all cathode materials used, LiMPO_4_ (M = Ni, Co, Mn, and Fe) as a phosphor-olivine-structured composite is as an excellent LIB cathode candidate with theoretical capacity of ∼170 mAh g^−1^ [33,34]. In this regard, LiFePO_4_ (LFPO) and LiMnPO_4_ (LMPO) have shown high efficiency as cathode-recharged performance LIBs [35,36]; however, these materials have limitations in terms of stable cyclability and rate capability induced by poor electronic and ionic conductivities. The LMPO structure displays higher energy density (4.1 V against Li/Li^+^) compared with LFPO (3.45 V against Li/Li^+^) [35–38]. This feature indicates that the LMPO materials can be easily used as a very powerful built-in electrode in EVs [37,38]. Supposing that fabrication of LMPO cathode can form super-hierarchical architectures, its high capacitive performance and high energy-density are expected to be improved significantly. Furthermore, a novel combination of environmentally friendly lithium-rich anode/cathode architects will create distinctively diffusible gateways for continuous electron/Li^+^ ion movements. To date, the low-cost fabrication and large-scale manufacture of LIB modules with prolonged charge/discharge timescales and maintaining high-energy density has not been achieved for the longest LIB-EV driving range.

**Scheme 1.**
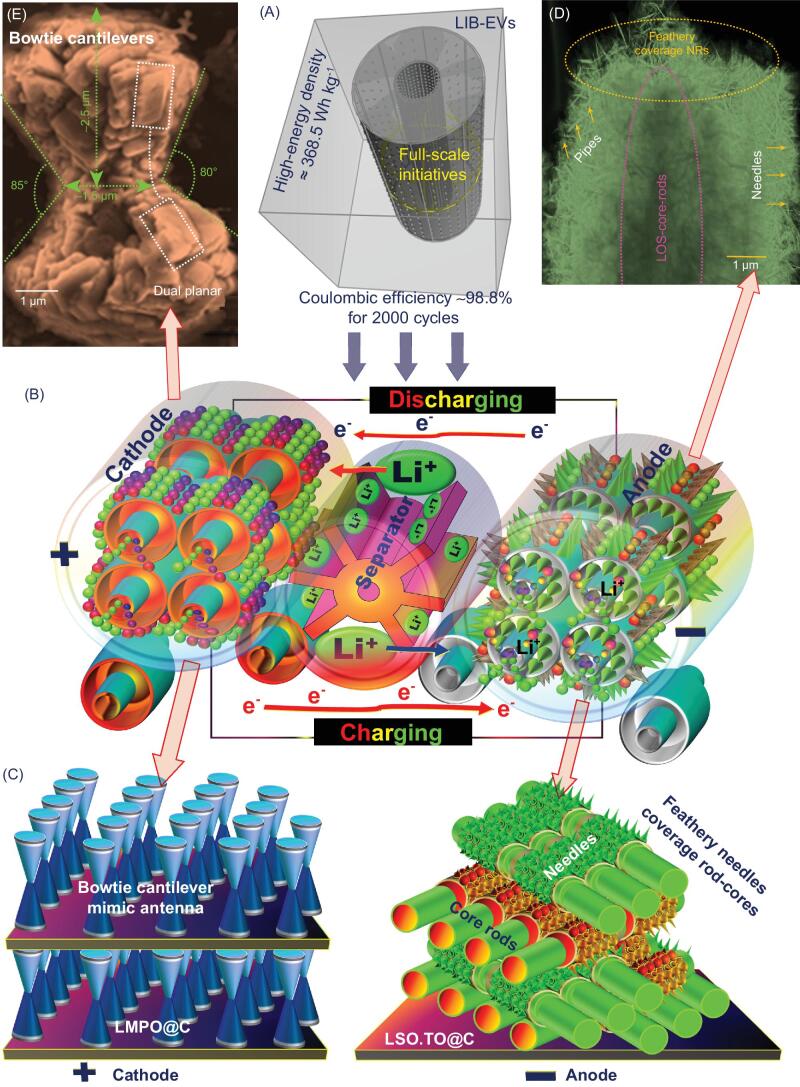


We report full-system LIB-CR2032 coin cell models for low-cost fabrication and high-energy density. The anode/cathode electrode surfaces are fabricated with super-hierarchical micro/nano architectures featuring anisotropic surface heterogeneity, accommodated multi-grooves and uniform ordering spaces, leading to long life cycle and high-energy density LIBs. The anodic LSO.TO@nano-C architectures are fabricated with core–pole rod skewers covered by vertical feathery needles. In addition, multilayer-stacked, dual-planar bowtie cantilevers mimicking antenna are specific to the cathodic LMPO@nano-C morphology. The key architectures of anode/cathode electrodes give the LIB models continuous in-/out-flow rates and fast transport pathways of Li^+^-ions even after recursively enumerable time-scale discharge/charge cycles (Scheme 1). This article provides evidence of hierarchical LMPO@nano-C (cathode)//LSO.TO@nano-C (anode) LIB-CR2032 coin cell models with excellent capacity storage, outstanding durability and Coulombic efficiency, and high-energy density, leading to a large-scale driving range of LIB-EVs.

**Scheme 1.** Schematic representation of cavity-packed circular simulation design of full-scale LIB-pouch models (A) that formed with usage of multiple rolls of coin cells and rolled out in a collar fashion of 18650-cylindrical or pouch models (B). (C) A well-packed, dense formation of Li-rechargeable battery coin cell with LSO.TO@nano-C-anode (5-layers/10-sides)//LMPO@nano-C-cathode (6-layers/10-sides). (A, Inset) Energy density and Coulombic efficiency for full-cell LSO.TO@nano-C//LMPO@nano-C design, at rate of 1C (0.2 A g^−1^) up to 2000 cycles, operated in potential range 2–4.5 V. (D, E) FE-SEM micrographs of anode (D) and cathode (E) recorded after multiple charge/discharge cycles.

## RESULTS AND DISCUSSION

### Fabrication of LSO.TO@nano-C anode and LMPO@nano-C cathode geometrics

**Figure 1.**
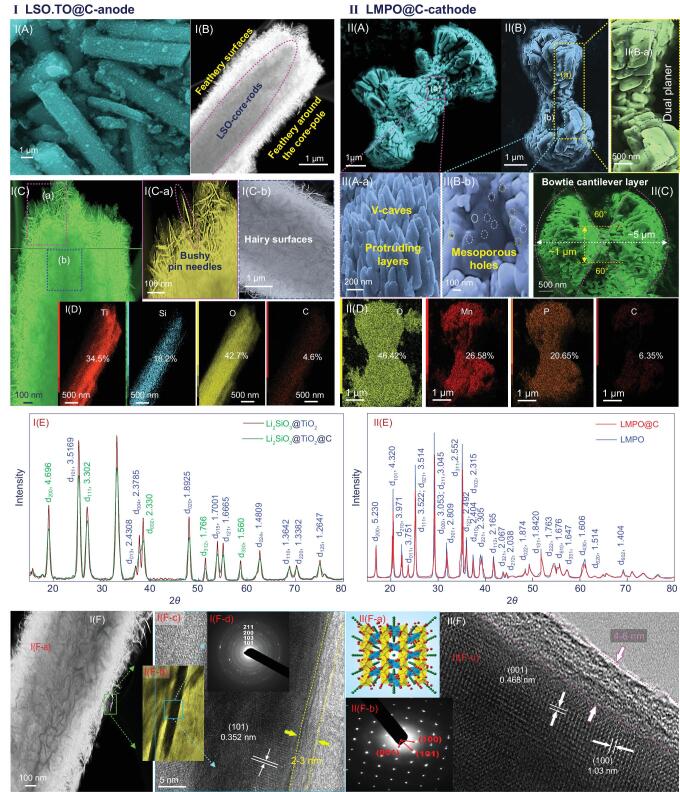
I(A–F) and II(A–F) Top- and side-views of FE-SEM (A–C), STEM-EDS elemental mapping analysis (D), XRD (E), HR-TEM and ED (F) images of heterogeneous architectures of LSO.TO@nano-C anode and LMPO@nano-C cathode. I(E) XRD patterns of LSO.TO (black-line) and LSO.TO@nano-C (red-line) and II(E) XRD patterns of LMPO (blue-line) and LMPO@nano-C (red-line) samples. I(F-a) STEM-HRTEM light-image, I(F-b) and I(F-c) high magnification STEM-HRTEM images, and I(F-d) ED lattice pattern of LSO.TO@nano-C anode. II(F-a) Crystal structure of [010] plane of olivine structure LMPO, II(F-b) ED lattice pattern and II(F-c) STEM-HRTEM image for LMPO@nano-C cathode. The microscopic edge surfaces of LSO.TO@nano-C/LMPO@nano-C indicate the formation of nano-carbon layer (with size of 2–6 nm range).

On the basis of primary LIB geometric controls, anodic LSO.TO@nano-C core–pole rod skewers vertically aligned with feathery needles on their upper surfaces provide remarkable functions for fabrication of half-cell LIB alternatives with better safety. In addition, control fabrication of LMPO@nano-C cathode with multilayer-stacked and dual-planar bowtie cantilever mimic antenna is achieved. The LMPO@nano-C antenna design is connected in the focal neck point to form a bi-head angle of acute scalene bi-triangle foldout ribs. The building block of the antenna cathode architect is the focal point for development of a low-cost fabrication route for LIBs with ever-decreasing discharge times (see Supporting information S2–S12). A protocol synthesis, time-dependent heterogeneous-particle diffusion, is used for fabrication of LSO.TO anode architects. During fabrication of LSO-rod–core/TO-feathery needle-shell structures, rapid formation of thermodynamically stable LSO core rod-like pole skewers is evident (Fig. 1). Along the formed 1D skewers, the bushy pin-needles of TO shell cover the LSO surfaces. In the antenna-like cathode fabrication route, phosphoric acid catalysis plays a key role in the growth domain of olivine LiMnPO_4_ architects. Also, ethylene glycol (EG) and ascorbic acid may assist in formation of layer-on-layer building blocks. The growth in lateral and longitudinal dimensions offers multilayer-stacked building-blocks and drives design of dual-planar bowtie cantilever mimic antenna-like morphology (see Supporting information S1, Scheme 1 and Fig. 1).

*Ex situ* carbon dressing of N-negative and P-positive electrode surfaces is controlled using a microwave-assisted approach. This *ex situ* carbon dressing protocol did not change the LSO-core@TO-shell wiry anode and LMPO-antenna cathode designs. The nano-C-bumps decorate the outer surface coverage architectures, leading to (i) generation of an actively assembled LSO.TO@nano-C bushy-needle anode, (ii) congruous mutation of a bowtie antenna LMPO@nano-C cathode, and (iii) formation on a large-scale and in/-output access-on-surface networks. The rigid dressing of nano-C-bumps along anode/cathode surfaces creates multi-voids and ridges for accommodation of electron/Li^+^ ions during discharge/charge cycles (Scheme 1).

In systematic LIB coin cell models (Scheme 1 and Fig. S1), the key influence of the architectural structure on the buildup design of half- or full-scale LSO.TO@nano-C//LMPO@nano-C LIBs is evident. The N- and P-electrodes designed in CR2032 coin cells with 3D modeling anisotropic super-hierarchy, multiscale architectures of anode/cathode and spatial component heterogeneity enable continuous in-/out-flow rates and fast transport pathways of electron/Li^+^ ions during charge/discharge cycles. Fabrication of LSO.TO@nano-C anode-based morphology with pin-hole feathery needles wrapping the upper surfaces of core–pole rod skewers would facilitate Li^+^ ion transport gates in multi-orientation directions (Scheme 1B-D-right). The structural stability of scalable feathery tissues along LSO.TO@nano-C-anode skewers would provide the N-electrode surfaces with the following:

- a branch of diffusional pocket-like storage to maximize the electron/Li^+^ ions and to diffuse into interior/exterior pipe channels to support the highly reversible capacity rate of LIBs;
- mitigation of the random distribution of Li^+^ ion in-/out-flow movement to attain maximum capacitance and power energy density within multiple charging/discharging cycles of LIBs;
- superior delithiation cycling stability and continuous Li^+^ ion-extraction process at LSO.TO@nano-C-anode surfaces at high potential values > 1.7 V, to improve safety issues of LIB-EV manufacturing.

Furthermore, the primary keys of LMPO@nano-C cathode bowtie antenna as architect-driven LIB geometrics would yield P-electrode cortex surfaces as follows (Scheme 1B-E-left):

- formation of the P-electrode surface with stacked bi-rectangular layer-by-layer sheets like cantilevers in dual planar bowtie antenna dipoles, offering multi-diffusible orientation systems;
- antenna configurations featuring a connected point at the focal neck, and forming a bi-head angle of acute scalene bi-triangle fold-out ribs, enabling creation of vast entrances and gates for electron/Li^+^ ion transport;
- reconfigurable, parabolic V-shaped cantilevers mimicking bowtie antenna design with double vertices and abundant surface defects/distortions, enabling multiple, and time-scale charge-discharge processes;
- P-electrodes with access-on-surface systems formed along ridges, V-shaped micro-cavities, edges, corners, and cortex faces, which support a large number of ultimate electron/Li^+^ ions (Scheme 1B);
- double-spooling vertices along 3D modeling of dual-planar bowtie antenna dipoles to minimize the intricate resistance distances of the P-electrode surface interaction and allow mounts of dense capacitance and well-defined electron deployment on the P-electrode surfaces.

### Characteristics of heterogeneous architectures along (anode/cathode) N- and P-electrodes

To investigate the anisotropic alignments of heterogeneous architectures along N- and P-electrodes, microscopic profiles, X-ray patterns, and textural and surface binding parameters were used (Fig. 1 and Supporting information S1–S10). Field-emission scanning electron microscope (FE-SEM) patterns (Figs 1A–C and S2) show the topographic morphology of super-hierarchal TO, LSO, LSO.TO, LSO.TO@nano-C anodes, and LMPO and LMPO@nano-C cathode architects. The topographic microscope patterns demonstrate evidence of the formation of core rod-like pole skewers and irregular, semi-spherical hairy/cotton surfaces of parent LSO and TO anodic architects (Fig. S2). The FE-SEM images of the hierarchical (LSO.TO and LSO.TO@nano-C) anode structures indicate core rod-like pole skewer coverage with scalable and mature feathery tissues. A mass of bushy pin-needle-like-wires allows for creation of sizable dimensions and multiple orientations in helical, latitudinal and longitudinal directions along anode surfaces (Scheme 1). Figure 1II(A–C) shows the LMPO and LMPO@nano-C cathode superstructure of flattened, multi-stacked and dual-planar bowtie cantilever layers (antenna-like design). The layer-by-layer building blocks form a bi-triangular sheet-like morphology, which is connected at one central vertex (Scheme 1). The LMPO@nano-C cathode architectures would form a wide range of dimensions, interconnected vicinities, surface interface heterogeneities, and micro-cavities along angularities, voids and boundaries. The dimensions along 3D modeling of dual-planar bowtie antenna dipoles are ∼2.5 μm planar half-height arm, ∼1.5 μm neck-joint span width and ∼70°–90° bracket angle, respectively. Such surface features would offer facile electron/Li^+^ ion pathways during charge/discharge processes, and Li^+^ ion accommodation and accumulation for long-term energy storage. Figures 1D and S4 give real evidence of a well-defined distribution of the atomic-scale contents of LSO.TO@nano-C and LMPO@nano-C composites in the configured morphologies of feathery needles, and wiry rods (anode), and bowtie cantilever-like antenna (cathode). Figure 1E shows evidence of formation of architecture crystals of LSO.TO and LSO.TO@nano-C, and LMPO and LMPO@nano-C composites (see Supporting information S3). The N_2_ adsorption–desorption isotherms (Fig. S5) confirm that the LSO.TO@nano-C and LMPO@nano-C anode/cathode electrodes are composed of high surface coverage and mesoscopic pore structures with typical and uniform cylindrical shapes. The sustainability in structure ordering, atomic-scale crystals, multi-reactive centering components/sites and textural parameters of N- and P-electrodes at high-temperature treatment creates exceptional value in fabrication of extremely stable, long-term LIB cycling (see Supporting information S7–S9).

The powerful architectures of LSO.TO@nano-C and LMPO@nano-C anode/cathode electrodes can be used as boosting surface models to create high performance half- or full-cell LIBs. Both electrodes are functionalized by multi-reactive centering atomic-scale crystal sites that associate with cortex faces, edges, corners, kinks and vertices. These electrode surface functions will drive unlimited mass transport of Li^+^ ions during charge/discharge cycles. Figures 1F and S2 show typical atomic-scale arrangements of crystal surface structures along the exposed crystal planes of LSO.TO@nano-C and LMPO@nano-C anode/cathode electrodes, evident from high-resolution transmission electron microscopy (HR-TEM) and scanning transmission electron microscopy (STEM) profiles, and electronic diffraction (ED) patterns. The microscopic patterns indicate well-defined distribution and configuration of structure content composites. Figures 1I(F-a)–(F-d) show the HR-TEM/STEM microscopic patterns of hierarchical LSO.TO@nano-C-anode morphology of feathery needles with top pin-hole-like vascular coverage core–pole rod skewers for high diffusion and electron/Li^+^-ion movement through interior channels and exterior surfaces. Multiple electron/Li^+^-ion pathway directions including zigzag, helical, spiral, latitudinal and longitudinal orientations can be created around abundant core–pole rod skewers. Figure 1I(F-c) suggests that the nanocarbon bumps are doped on upper-zone ridge surfaces. The ED pattern of LSO.TO@nano-C demonstrates well-developed fringes with dominant [111]/[101] planes, referring to pure LSO rod-core and anatase TO shell (Figure 1I(F-d)). Figure 1II(F-a)–(F-c) shows the 3D crystal structure, ED pattern and HR-TEM profile along the [010] plane of cathodic LMPO@nano-C dual planner bowtie-like antenna crystals. The d-spacing values of 0.46 and 1.03 nm of the neighboring lattice fringes are in agreement with (001) and (100) planes, respectively (Figure 1II(F-c)). This ED pattern illustrates that the LMPO@nano-C single crystal structures are configured in large-domain atomic-scale arrangements along the [010] direction. The dominant [010]-LMPO@nano-C bowtie cantilever antenna indicates an *ac* plane as an actively exposed site for heavily loaded electron/Li^+^ ion coverage. This *ac* plane is the preferable facet orientation to maintain continuous Li^+^-ion diffusion along the active site-exposed *Pnma*-orthorhombic structures of LMPO@nano-C cathode [34,39]. The top-zone-views of out-plane [010]-LMPO@nano-C antenna surfaces and [111]/[101]-LSO.TO@nano-C feathery needles highlight the carbon coverage layers. Hierarchical architectures of anode/cathode electrodes with nano-scale ridges, caves, channels and open-end pipes can affect fabrication of half- and full-system CR2032 coin cell models with extraordinary and multi-directional diffusions of Li^+^ ions during lithiation/delithiation.

### Formulation of LIB designs with half- and full-system CR2032 coin cell models

Along with fabrication of LIB coin cell designs and models, we explored the incentives and keys of anode/cathode architectures with anisotropic surface heterogeneity, multi-component reactive plane sites, dimension scales and vacancies, and composite textures on their electrochemical performance. The primary architect-driven electrode geometrics using anodic LSO.TO@nano-C core–pole rod skewers, and cathodic LMPO@nano-C bowtie cantilever antenna lead to a low-cost fabrication route for high-energy density full LIBs. The designed anode N-electrode is used to build anodic half-cell LIBs using a wide range of TO-anatase-cotton-shape, LSO-core-pole rods, LSO.TO and LSO.TO@nano-C architectures. Also, the LMPO and LMPO@nano-C-cathode electrodes are used to design cathodic half-cell LIBs. Among all anodic and cathodic electrodes, we formulated full-scale LIB-CR2032 coin cell models from LSO.TO@nano-C and LMPO@nano-C anode/cathode (N/P) electrodes. In the pattern of full-system LIB-CR2032 coin cell models, we modulated the N/P loading amount to maximize power energy density, to store the electrons in better safety models, to diffuse Li^+^ ions along in-/out-flows, and to boost the discharge-specific capacitance of full-scale LIBs. Aiming to scale up manufacturing of LIB cell modules, a wide range of full-system LIB-CR2032 coin cell models can be modulated in packing layers of pouch-type modes. Our subsequent studies show that fabrication of LIB coin cell designs, and large-scale models would lead to ever-decreasing discharge and harmonic movements of electron/Li^+^ ion during lithiation and delithiation cycling.

### Super-anodic hierarchy half-cell LIBs using TO, LSO, LSO.TO and LSO.TO@nano-C N-electrodes

**Figure 2.**
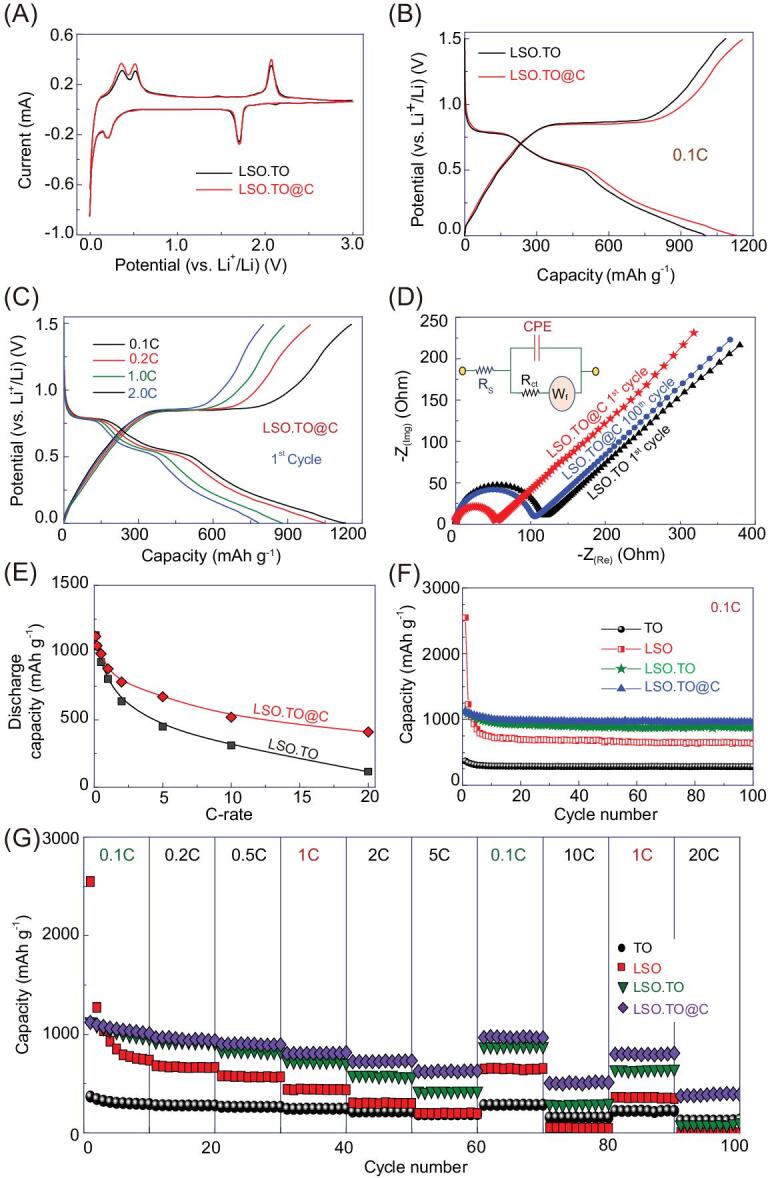
(A) CV results of the electrochemical effectiveness of anodic CR2032 coin-type half-cell LIBs. Typical cyclic voltage patterns of the LSO.TO (black-curve) and LSO.TO@nano-C anodes (red-curve). (B) The charging/discharging voltage paradigms of first cycle LSO.TO and LSO.TO@nano-C anodic half-cells at a current rate 0.1C. (C) Charge-discharge voltage profiles of first cycle LSO.TO@nano-C anode half-cell at C-rates. (D) EIS results for LSO.TO and LSO.TO@nano-C half-cell anodic electrodes. (Inset of d) The equivalent circuit shows agreement with the impedance results. (E) Behavior of specific discharging capacity versus C-rates ranged from 0.1C to 20C for half-cell LSO.TO@nano-C anode. (F) Cycling performance stability of TO, LSO, LSO.TO and LSO.TO@nano-C anodes in half-cell CR2032 coin cells determined at C-rate of 0.1C, and after 100 cycles. (G) Performance of rate capability among all tested TO, LSO, LSO.TO and LSO.TO@nano-C anodes. Electrochemical measurements for TO, LSO, LSO.TO and LSO.TO@nano-C anodic half-cells are operated at a wide range of voltages (1.0–3.0 V), (0.1–2.0 V), (0.1–1.5 V) and (0.1–1.5 V), respectively.

A wide range of architectures of TO cotton, LSO nanorods and LSO.TO and LSO.TO@nano-C core–pole rod skewers is used to fabricate anode half-scale LIB-CR2032 coin cell models. The key LIB design based on the ordered sets of a series of N-anode coin cells is studied. Figures S1−S10 and S1−S11 show the electrochemical performance of TO and LSO as anode half-cell LIBs. To modulate the spatial anodic electrode in potential half-cell LIBs, we studied cyclic voltammogram (CV) measurements for several TO, LSO, LSO.TO and LSO.TO@nano-C anode N-electrodes. Figure 2A shows the CVs for LSO.TO and LSO.TO@nano-C-anode (N) electrodes. The CV outcomes indicate that the capacity of a hierarchically matured LSO.TO negative anode electrode is determined by the building block of TO hairy/cotton anatase phase along the LSO core–pole rods. The CV curve of the anodic LSO.TO@nano-C core–pole rod skewers reveals a remarkable electrochemical current enhancement at a 0.1 mV s^−1^ potential compared to that of the LSO.TO (black-color curve). This finding indicates the massive effectiveness of hierarchical architectures of LSO.TO@nano-C core–pole rod skewers in power-driven LIB-half-cell anodes.

The LSO.TO@nano-C anode half-cell LIBs show outstanding performance of charging/discharging cycles compared with LSO.TO-based electrodes. Figure 2B illustrates the charge-discharge voltage profiles of first cycle LSO.TO and LSO.TO@nano-C anodic half-cells at a current rate of 0.1C. The result shows the effect of dressing the nano-C-bumps along the core–pole rods (LSO.TO) in enhancement of cycling stability. As shown in Fig. 2B, the LSO.TO anode exhibits charging and discharging specific capacities of approximately 1130 and 1120 mAh g^−1^, respectively, with Coulombic efficiency of 91.6% in the first cycle. In turn, the charge and discharge specific capacities of the LSO.TO@nano-C-anode are 1158 and 1131 mAh g^−1^, respectively, with superior Coulombic efficiency of 97.6% in the first cycle. This finding indicates that the dressing of nano-C-bumps along the LSO.TO@nano-C surfaces not only enhances their conductive or electronic features, but also acts as a protective surface layer of the anode electrode. This nano-C-coverage layer may suppress the atomic-scale de-localizations (i.e. in-/out-crystal geometric structures) or symmetric phase decomposition of LSO.TO@nano-C composites. The long-standing stability of the primary architect-modal LSO.TO@nano-C geometrics constrains the silicon (Si) volume expansion-exfoliation along the coherent atom-surface and phase-modulated crystal structures of anodic core–pole rod/feathery needles. Consequently, the LSO.TO@nano-C key structure in buildup design of anode half-cell LIBs becomes evident, boosting the stability of time-scale discharge/charge cycles, and improving the Coulombic efficiency, especially for the first cycle.

To investigate the effect of the current rates on cycling performance of the LSO.TO@nano-C anode half-cell LIBs, the charge-discharge voltage profile of the first cycle LSO.TO@nano-C anode half-cell at different C-rates (0.1–2C) is shown in Fig. 2C. Our results show evidence that the first cycle of LSO.TO@nano-C anode attains ∼100% Coulombic efficiency with negligible decrease in its value even at high C-rate (i.e. 2C-rate). The long-standing Coulombic efficiency of the anisotropic LSO.TO@nano-C anode electrode architecture at high C-rates is the result of superior interfacial electrolyte/anodic-electrode film phases. The interfacial phases are homogenously formed throughout (i) the exterior surface topography (i.e. V-caves, pipes, cylindrically shaped meso-holes, feathery needles, and bushy canals and grooves), (ii) the upper-edge surface ridges, and (iii) along the interior vacancy spaces. The ideal Coulombic efficiency and the great capacity retention of the LSO.TO@nano-C anode half-cell LIBs indicate the hotkey stability of anisotropic composite textures, centrally mounted heterogeneity surfaces and multi-reactive centering components/sites for long-life battery cycles [40–47].

To explore the key functions of anode half-cell LIBs, we studied the behavior of specific discharge capacity (mAh g^−1^) for TO, LSO, LSO.TO and LSO.TO@nano-C anode half-cells versus the current C-rates (i.e. from 0.1 to 20C), (see Figs S11e, f and 2E). Moreover, Fig. 2F illustrates the cycling performance of TO, LSO, LSO-TO and LSO-TO@nano-C electrodes as built-in anode half-cells at 0.1C. Among all anode-half-cell systems, the built-in LSO-TO@nano-C anode shows excellent electrochemical performance compared with pristine TO, LSO and LSO-TO anodes. The TO-anode exhibits a high retained capacity of 76.55% (1.0–3.0 V) after the 100th cycle and low discharge capacity value of 282.38 mAh g^−1^ out of its first-cycle capacity of 368.89 mAh g^−1^. The LSO-anode shows poor cycling behavior with 24.76% retained capacity from the initial discharge capacity of 2548.28 mAh g^−1^ after 100 cycles and at 0.1–2.0 V. The LSO.TO anodic half-cell (0.1–1.5 V) attained 78.5% (i.e. 879 mAh g^−1^ of its initial capacity 1120.3 mAh g^−1^) after 100 cycles. The LSO.TO@nano-C-anode half-cell LIBs featured an outstanding retention capacity of 84.8% and enhanced cycling performance (i.e. 959 mAh g^−1^ of the first-cycle capacity 1131.78 mAh g^−1^) after 100 cycles.

A set performance of the anode retention of discharge specific capacity within cycles was studied to investigate the rate capability. This study reveals the effective super-anodic hierarchy on the contiguous charge/discharge cycles, as evidenced from Fig. 2G. To choose the super-anodic hierarchy in LIB designs, intensive comparisons were made of TO, LSO, LSO.TO and LSO.TO@nano-C anode half-cells. The LSO.TO@nano-C anode indicates a higher rate capability than that of TO, LSO and LSO.TO anodes at various rates, according to the following C-rate sequence patterns of 0.1, 0.2, 0.5, 1, 2 and 5C. These C-rates return back to 0.1 and 10C, and then to 1 and 20C, showing a high rate capability. Figure 2G provides evidence of the significant effect of the anisotropic surface heterogeneity of the LSO.TO@nano-C anode on the long-period cycling stability. An excellent retention of discharge specific capacities of the LSO.TO@nano-C anode is found at approximately 1131, 940, 895, 807, 730, 629, 510 and 390 mAh g^−1^, at rate patterns of 0.1, 0.2, 0.5, 1.0, 2.0, 5.0, 10 and 20C, and at different cycling numbers of 1st, 20th, 30th, 40th, 50th, 60th, 80th and 100th cycles, respectively. LSO.TO@nano-C showed the best rate capability compared with that of other tested anodes (i.e. TO, LSO and LSO.TO). Results also suggest that the specific discharge capacity of LSO.TO@nano-C anode is fully recovered at the reduction of charge-discharge rate pattern from 5C to 0.1C and from 10C to 1C, respectively. This finding indicates the excellent rate performance of the LSO.TO@nano-C half-cells. The specific capacity of super-anodic LSO.TO@nano-C hierarchy is 390 mAh g^−1^ at 20C. Together, the results suggest that the LSO.TO@nano-C anode has substantially outstanding specific capacity of N-electrode compared with the graphite capacity (i.e. theoretical value of 372 mAh g^−1^) at 1C [10,11].

Electrochemical impedance spectroscopy (EIS) exhibits Nyquist plots for TO, LSO, LSO.TO and LSO.TO@nano-C anodic electrodes (see Fig. S11c, d, respectively). The semicircle and slanted line are shown at high- and low-frequency regions, respectively, as evidenced from the Nyquist graphs. In addition, the equivalent circuit is investigated according to EIS results, as shown in the inset of Fig. 2D [42,43]. The charge transfer resistance (R_ct_) values for TO, LSO, LSO.TO and LSO.TO@nano-C anodes are 182, 201, 122 and 54 Ω, and 283, 490, 193 and 104 Ω after the first and 100 cycles, respectively. The R_ct_ value of LSO.TO@nano-C composite anode is lower than that of the pristine TO, LSO and LSO.TO composites after the first cycle or even after 100 cycles. The R_ct_ values for the first and 100th cycles are in the following order: LSO.TO@nano-C < LSO.TO< TO< LSO. Among all N-electrodes of TO, LSO and LSO.TO anodes, the LSO.TO@nano-C anode shows a high Li^+^ ion diffusion ability in the order of LSO.TO@nano-C > LSO.TO> TO> LSO. The R_ct_ value indicates that the super-anodic LSO.TO@nano-C hierarchy exhibits (i) a minimum transfer resistance compared with other anode architects, (ii) excellent transfer kinetics of electron/Li^+^ ion during the lithiation/delithiation process and (iii) high electronic transport and electrical conductivity surfaces. These EIS results further support the outstanding cycling performance of the super-anodic LSO.TO@nano-C hierarchy. In general, our anodic half-cell LIBs would lead to low-cost fabrication avenue, ever-decreasing discharge timescale and maintaining high-energy density that may improve the long-driving range of EVs.

### Half-cell LMPO and LMPO@nano-C cathode architectures

**Figure 3.**
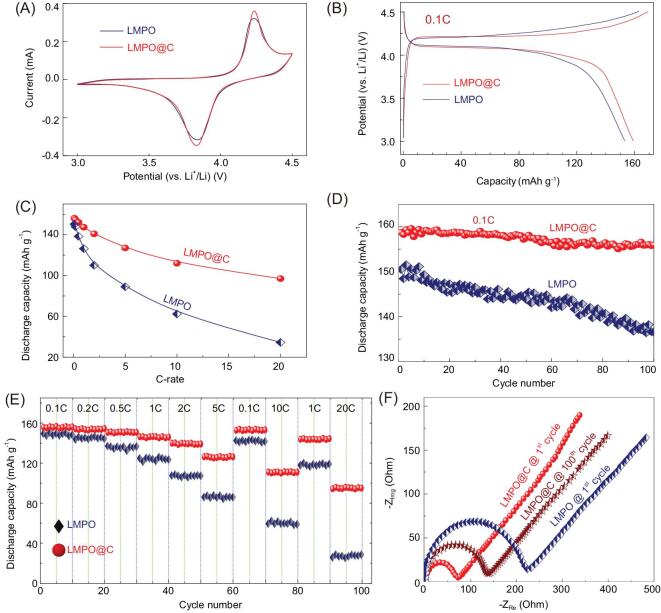
(A) First cycle voltammograms of the electrochemical performance of the LMPO (black-curve) and LMPO@nano-C (red-curve) cathode half-cell LIB-CR2032 coin-type at 0.1 mV s^−1^. (B) The charging/discharging voltage paradigms of half-cell LMPO and LMPO@nano-C cathodes investigated at C-rate of 0.1C. (C) Behavior of specific discharge capacity in mAh g^−1^ versus current C-rates from 0.1C to 20C for half-cell cathodes. (D) Cycling performance (stability) for cathode 2032 coin-type half-cell LIBs at a rate of 0.1C, and after 100 cycles. (E) Performance of rate capability among tested LMPO and LMPO@nano-C cathodes. (F) EIS results for half-cell LMPO and LMPO@nano-C cathodic electrodes. Overall electrochemical patterns for half-cell LMPO and LMPO@nano-C cathodes are investigated at 25°C, and at a wide range of voltages of 3.0–4.5 V.

The electrochemical performance of the half-cell LMPO@nano-C and LMPO cathode LIB-CR2032 coin cell models (Fig. 3) was studied. The cathode half-cell LIB is designed with specific surface features as follows (i) re-configurable, parabolic cantilever mimic bowtie antenna design, (ii) accommodated multi-grooves and mounts of nano-scale ordering vacancies and spaces and (iii) double site vertices and abundant surface ridges formed along V-shaped caves, edges, corners and cortex faces. The cathode dimensions, interconnected vicinities, surface interface heterogeneities and micro-cavities along angularities, voids and boundaries lead to stable inflow rates of abundant Li^+^ ion diffusions during multiple, and time-scale charge-discharge processes. A key formulation of P-electrode half-scale LMPO@nano-C and LMPO (cathode) LIB-CR2032 coin cell-type was designed using 10 μm-Al foil electrode under specific protocols for electrochemical measurements (see Supporting information).

Typical CV (Fig. 3A) results confirm that the built-in LMPO@nano-C cathode demonstrates a remarkable electrochemical current at a sweeping rate 0.1 mV s^−1^ compared with that of LMPO cathode-based half-cell LIBs. The CV curves show two peaks of oxidation (anodic delithiation) and reduction (cathodic lithiation) signaling for LMPO and LMPO@nano-C architect cathodes at 4.24 V/3.84 V. The significant peaks are attributed to the Mn^3+^/Mn^2+^-insertion/extraction with Li^+^ ion movement along the LMPO olivine crystal structure. Figure 3B illustrates the charging and discharging cycles of LMPO and LMPO@nano-C half-cell cathodes. The LMPO and LMPO@nano-C cathode cells are practically powered at charge voltage pattern to 4.5 V at 0.1C that maintained at 4.5 V for 1 hour, and then discharged to 3.0 V at 0.1C, respectively. The half-cell LMPO@nano-C cathode LIB exhibits higher specific capacity than that of the LMPO antenna cathode. At first cycle (0.1C), the antenna LMPO@nano-C cathode reveals high discharge capacity (159.5 mAh g^−1^) compared with that of LMPO (153.5 mAh g^−1^) electrode (Fig. 3C). Figure 3D shows superior capacity retention at the long-term cyclability (i.e. stability) of the LMPO@nano-C cathode. Results indicate that only 89.5% efficiency (i.e. equivalent to 137 mAh g^−1^) of the original capacity of 153.5 mAh g^−1^ is retained after 100 cycles for the LMPO cathode (0.1C). In turn, the LMPO@nano-C cathode maintains 99.2% efficiency (i.e. equivalent to 157.8 mAh g^−1^) of the primary capacity ∼159 mAh g^−1^. Furthermore, at a high C-rate of 20C, the LMPO@nano-C cathode exhibits higher rate capability than that of LMPO cathode P-electrode.

To explore the long-term cycle stabilization of cathode half-cell LIBs, the rate capability is a significant factor to manufacture LIBs with the high energy density needed for EVs and PHEVs. Figure 3E illustrates the charge/discharge cycle performance for LMPO and LMPO@nano-C cathodes at 0.1–20C along the 10 cycles. For instance, at 20C and after 100 cycles of repeated Li-intercalation, the LMPO@nano-C cathode exhibits a remarkably reversible discharge capacity (∼101 mAh g^−1^), while the LMPO cathode retains low capacity (28.4 mAh g^−1^). The Nyquist plot of LMPO@nano-C (Fig. 3F) shows a semicircle with a small diameter that reflects the lower charge transfer resistance value than that of LMPO cathode architects. The finding indicates that the micro-, nano-hierarchical LMPO@nano-C cathode has high electronic conductivity and excellent electron/ion transfer kinetics during lithiation/delithiation and across the electrode/electrolyte film interfaces. Indeed, the key dressing of the nano-C-bumps along the LMPO cathode cuticles has enhanced Li^+^ ion diffusion significantly.

Overall, the LMPO@nano-C electrode modulated in half-cell LIBs exhibits high Li^+^ ion specific capacity and excellent rate capability performance. These properties are attributed to the following micro-, nano-hierarchical architecture key factors:

- the large diversity of regularly morphological LMPO@nano-C shapes such as flattened, dual planar and multi-stacked bi-rectangular sheets in a bowtie antenna hierarchy;
- stacked layer-by-layer sheets formulated in the dipole antenna design, which are key to micro- and nano-scale building blocks of cantilevers;
- the multi-reactive centering components/sites in perfect crystal orientation created along bi-triangular micro-V-shaped cavity antenna, and between a number of connectivity with five vertices and six edges oriented in bowtie antenna;
- a wide range of dimensions and orientations, interconnected vicinities, surface interface heterogeneities and micro-cavities along angularities, voids and boundaries as a result of formation of bowtie antenna building blocks.

### Configurations of large-scale, full-system LIB-CR2032 coin cell models

This section describes key parameters of LIB-design configurations to control full-system LIB-CR2032 coin cells, and large-scale pouch-type models. We demonstrate diverse LIB models for achieving excellent rate capability and charging/discharging capacity, and high volumetric energy density. We studied full-scale LIB model reliability and validity based on correlation control of the tradeoff between improved safety and maintenance of high energy density. Also, we investigated the influences of preferable LSO.TO@nano-C anode//LMPO@nano-C-cathode electrodes designed with (3D) modeling superhierarchy, multiscale architectures and anisotropic surface heterogeneity on the ordered set configurations of pouch-type models.

To calculate the cell-energy density of full-scale LIBs, the stacked layers of LSO.TO@nano-C N-anode//LMPO@nano-C P-cathode-CR2032 coin cells were designated in pouch-LIB-types (see Supporting information S15). A key model of full-scale LSO.TO@nano-C//LMPO@nano-C (P-anode//N-cathode) pouch LIBs was achieved by rational loading of actively centering components on both negative 8 μm-Cu and positive 10 μm-Al electrodes, respectively (i.e. the N/P balancing capacity ratio). The multiple stacked layers of incorporated amounts of LSO.TO@nano-C core–pole rod skewers (N-electrode anode)//LMPO@nano-C dual-planar bowtie antenna (P-electrode cathode) are five-//six-layers, and along 10 sides. These five-anode//six-cathode layers were used to decorate 8 μm-Cu- and 10 μm-Al-foil electrodes, respectively. Fine-tuning of the (N:P)_cap_ ratio was crucial to controlled experiment sets and to offer a similar discharge specific capacity along N-, and P-electrodes (i.e. the (N:P)_cap_ ratio is 1:1) (see Supporting information S14–S16).

Furthermore, the volumetric energy density of the 18650 cylindrical-shaped LSO.TO@nano-C//LMPO@nano-C full-scale LIB-model is higher than that of pouch LIB-types (see Supporting information S17). The volumetric energy density also has preferable values for controlling manufacture of large-scale LIB-EVs. Our experimental calculation shows that the areal discharge capacities of LMPO@nano-C-cathode and LSO.TO@nano-C-anode electrodes are 1.24 Ah cm^−2^ and 1.325 Ah cm^−2^, respectively. The areal discharge capacity of N- and P-electrodes is determined at a specific (N:P)_cap_ capacity ratio of 1.07:1. This rational (N:P)_cap_ value is controlled to produce an optimal tradeoff stacked pouch LIB-model (see Supporting information S15–S16).

**Figure 4.**
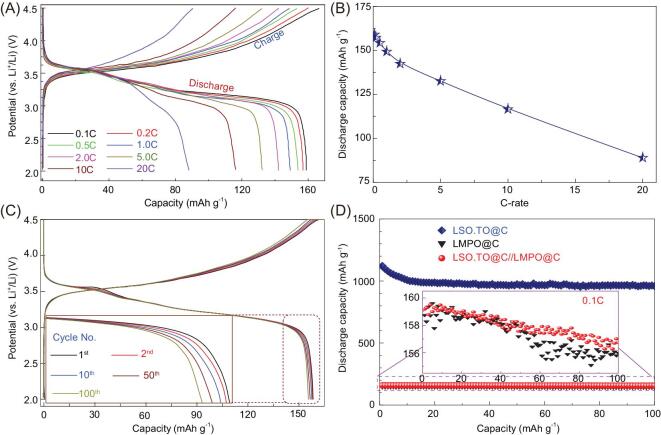
Electrochemical performance of full-system LIB-CR2032 coin cell models designed by LSO.TO@nano-C anode and LMPO@nano-C cathode electrodes, respectively. (A) First-cycle charging/discharging voltage paradigms at different C-rates of 0.1C–20C. (B) Behavior of specific discharging capacity density versus C-rates ranging from 0.1C to 20C. (C) Electrochemical charge-discharge voltage profiles at different cycles up to 100 cycles, at 0.1C. (D) Cycling performance (stability) at different cycles up to 100 cycles, at 0.1C. Overall electrochemical patterns for half-cell full-cell models are investigated at 25°C and at a wide range of voltages of 2.0–4.5 V.

Figure 4A shows the typical first cycle of charging/discharging voltage paradigms of full-scale LSO.TO@nano-C//LMPO@nano-C LIB-CR2032 coin cell model cycles recorded at 0.1, 0.2, 0.5, 1, 2, 5, 10 and 20C, and at voltages between 2.0 and 4.5 V. Our finding provides evidence that the average working potential of the LSO.TO@nano-C//LMPO@nano-C full-scale battery is 3.45 V. This working potential value results in outstanding energy density for the cathode P-electrode (Wh kg^−1^) of ∼516.5 Wh kg^−1^ at 1C (see Supporting information S13). In the practical fabrication of LIB, the configuration of ordered-sets of full-scale cathode//anode pouch LIB-models can be controlled under optimized loading amounts (mass/coverage area, mg/cm^2^) of 3.9 and 13.8 mg/cm^2^ for the LMPO@nano-C-cathode and LSO.TO@nano-C-anode electrodes, respectively. The optimal accuracy of the estimated cathode mass-loading fraction, as individual electrode components forming a LIB-CR2032 coin cell model, is about 46% (see Figs S12 and S13). Accordingly, the specific energy density of the LSO.TO@nano-C//LMPO@nano-C full-scale LIB is 237.6 Wh kg^−1^. This suggests that our full-scale LIB is promising for long-term EV-driving range with excellent power density (see Supporting information S12–13).

Figure 4B illustrates rate capability of LSO.TO@nano-C//LMPO@nano-C full-scale LIBs determined at current density values between 0.1C and 20C. Figure 4B reveals that the values of discharge capacities decrease with increasing C-rate (i.e. from 0.1C to 20C). The LIB full-cell maintains a discharge capacity of 89 mAh g^−1^ at 20C from its initial value. The obtained results indicate that the rate capabilities retention of the coin cell LIBs at 1C, 5C, 10C and 20C are 94%, 83.4%, 73.3% and 56% compared to the capacity density at 0.1C, respectively. Figure 4C reveals that the full-scale coin cell LIB capacities in the first cycle charging/discharging voltage paradigms at 0.1 C are 165 and 159 mAh g^−1^, respectively. These capacity values are equivalent to attaining 96.4% of the initial Coulombic efficiency. From the second cycle up to 100 cycles of these formulated LIB CR2032 coin cells, the Coulombic efficiency was found to be ∼100%. As a result, the small quantity loss of irreversible capacity during the first cycle is probably a result of electrolyte decomposition and growth of SEI-layers on the architect LSO.TO@nano-C anode surfaces. Figure 4D exhibits the cycling effectiveness and stability of the full-cell LSO.TO@nano-C//LMPO@nano-C LIB compared with the anode- or cathode-based half-cells at a C-rate of 0.1C and at different cycle numbers from the 1st to the 100th cycles.

**Figure 5.**
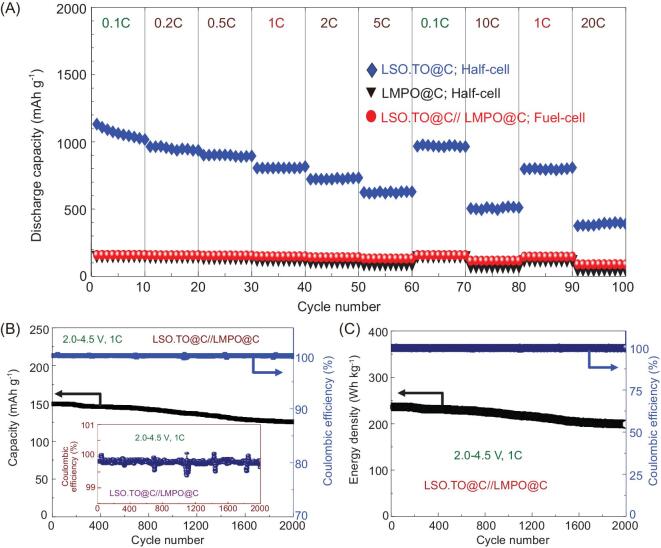
(A) Performance and behavior of the rate capability for LSO.TO@nano-C half-cell anode, LMPO@nano-C half-cell cathode and full-cell LSO.TO@nano-C (anode)//LMPO@nano-C (cathode) designs. (B) Long-range cycling effectiveness and Coulombic efficiency for full-scale LIBs at a rate of 1C up to 2000 cycles and magnified views of the potential Coulombic efficiency (inset) at 1C. (C) Energy density and Coulombic efficiency for full-cell models at a C-rate of 1C and after 2000 cycles. Overall electrochemical patterns for half-cell full-cell models are investigated at 25°C and at a wide range of voltages of 2.0–4.5 V.

Figure 5A shows the outstanding rate capability of the hybrid LSO.TO@nano-C//LMPO@nano-C full-cell at different C-rates. The charge-discharge cycling performance recorded at 0.1C (10th cycle), 0.5C (25th cycle), 1C (40th cycle) and 5C (60th cycle) and returned to 1C (90th cycle) and 20C (100th cycle), indicated discharge capacities of 157.8, 154.4, 148.5, 132.5, 146.7 and 88.8 mAh g^−1^, respectively. Taken together, the anisotropic alignments of heterogeneous architectures in full-scale LSO.TO@nano-C//LMPO@nano-C LIBs enable excellent long-term cycling effectiveness and stability.

Figure 5B reveals the Coulombic efficiency and cycling effectiveness of LSO.TO@nano-C//LMPO@nano-C LIBs at 2.0–4.5 V and 1C rate. The LSO.TO@nano-C//LMPO@nano-C full-cell attains 89% of the original capacity after 2000 cycles, and ∼99.89% average Coulombic efficiency. To evaluate the energy density, we carried out cycling performance of built-in full-cell LSO.TO@nano-C//LMPO@nano-C LIBs at a high rate of 1C up to 2000 cycles. Figure 5C shows that the LSO.TO@nano-C//LMPO@nano-C full-cell LIBs have superior cycling stability and high energy density of 237.6 Wh kg^−1^ with excellent durability. The full-cell LIB design maintains specific capacity of ∼89.8% within 2000 cycles and average Coulombic efficiency of ∼100% at 1C rate. This significant finding is technically the first of an energy density as demanded for improvement in the driving range of EVs. Indeed, our full-cell LIBs could configure scaled-up commercial requirements of modest EV demands of high energy density and specific capacity, better safety and inexpensive capital with long-term cycle-life period (Scheme 1).

**Scheme 2.**
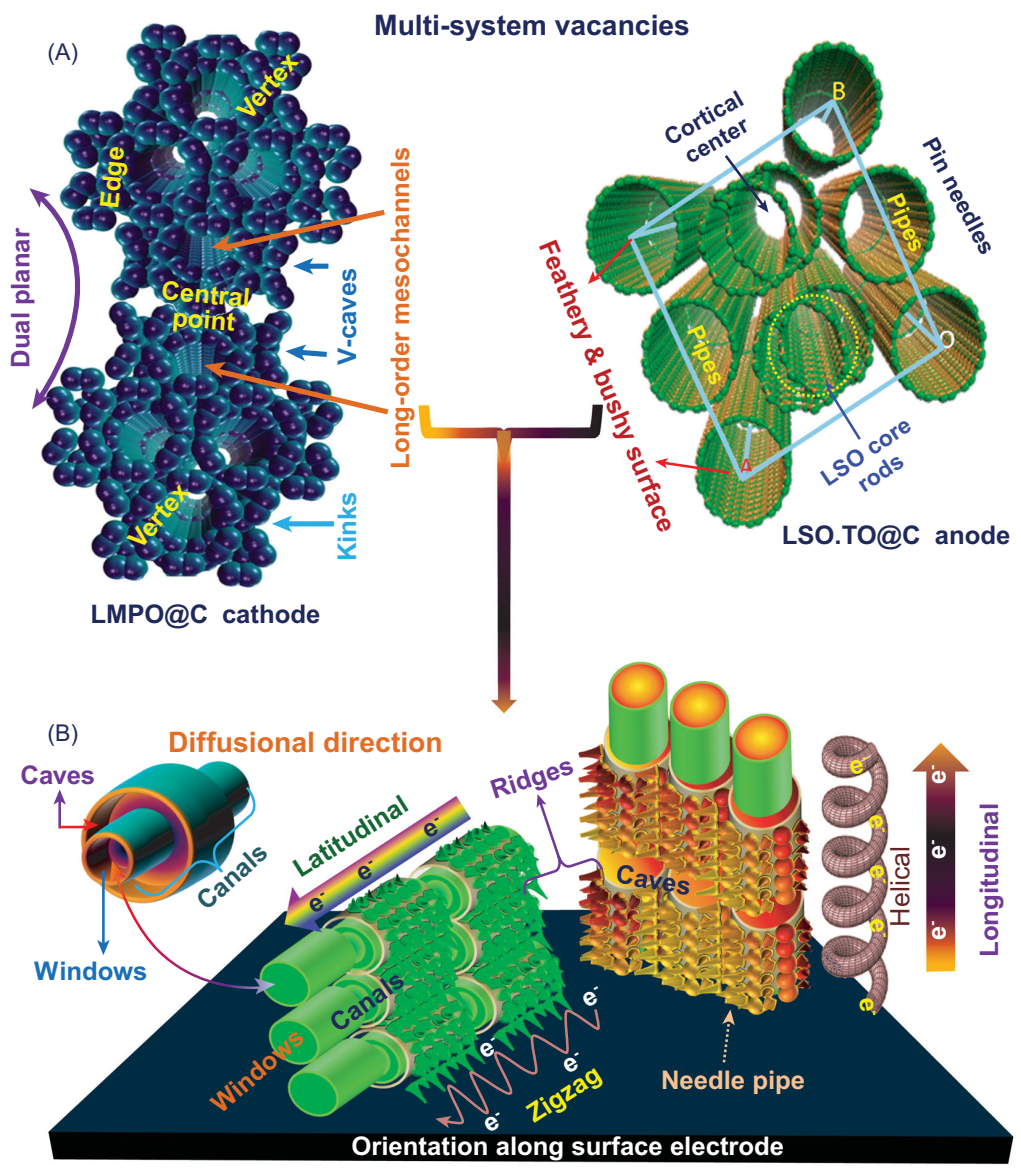
(A) 3D modeling of multi-system vacancies and (B) 3D directional orientations along super-hierarchal LSO.TO@nano-C (anode) and LMPO@nano-C (cathode) design architectures and their morphological configurations in 3D-planes after multiple charge/discharge cycles of the half- and full-cell LIB models.

To investigate the effectiveness of the reconfigurable design and super-hierarchal structure key to buildup design of LIB-full-cell LSO.TO@nano-C//LMPO@nano-C architects, we stimulated 3D modeling of multi-system vacancies after multiple charge/discharge cycles of the full-cell LIB design (Fig. S18 and Scheme 2). The projection design shows evidence of long-standing stability of heterogeneous LSO.TO@nano-C and LMPO@nano-C electrode architectures with significant features of (i) multi-reactive centering components/sites (i.e. cortex faces, edges, corners, kinks, vertices or central points), (ii) mounts of nano-scale-ordering vacancies and spaces (i.e. V-shaped caves, pipes, cylindrically shaped meso-holes, needles, and feathery and bushy canals) and (iii) a wide range of dimensions and orientations, interconnected vicinities, topographic surface heterogeneities and micro-cavities along angularities. Retention of the structural electrode features facilitates continuous and fast Li^+^-ion in-/out-flow rates, and multiple transport pathways for electrons/Li^+^ ions after multiple charge/discharge cycles. Scheme 2 shows the stability of full-system LIB-CR2032 coin cell models, indicating excellent time-scale reversibility. Despite the multiple cycles (∼2000) of the full-cell design, the multi-diffusible orientation systems, 3D modeling super-hierarchal architectures, morphological building blocks and anisotropic surface heterogeneity of multi-reactive system components offer multi-gateway migrations, and unlimited mass transport of Li^+^ ions. These multi-diffusible access-on-surface systems are vital for recursively enumerable time-scale discharge/charge cycles without blocking assignments (Scheme 2A and B) [45–50].

Scheme 1(D, E) shows FE-SEM micrographs of reusable LSO.TO@nano-C, LMPO@nano-C after charge/discharge cycles. The microscopic patterns indicate the structural stability of the anode and cathode architectures within multi-cycles. The cycled LSO.TO@nano-C morphology attains the interior core-pole rods covered by feathery and bushy needles (Scheme 1D). The cathodic LMPO@nano-C microscope pattern shows evidence of retention of layer-by-layer bi-rectangular sheets formulated in a dipole antenna design, despite the overlapping protruding layers after ∼1000 cycles (see Supporting information S18) [50]. Our microscopic findings present evidence of retention of the primary cathode/anode structures, orientations and dimensions. For instance, the cycled LMPO@nano-C cantilever bowtie antenna is attained with micro-V-shaped cavity dimensions and angularities with planar half-height arm, neck-joint span width, and bracket angle of ∼2.5 μm, ∼1.5 μm, and ∼70°–90°, respectively. In general, our findings indicate that the key design of full-system LIB-CR2032 coin cell configurations enables retention of electron/Li^+^ ion pathways along the architectural-design surface coverage without vortex surface hindrances [46,49–51]. Stability of access-on-surface and transport gateways onto cycled anode/cathode electrodes would create recursively enumerable and continuous diffusion systems of electrons/Li^+^ ions, excellent capacity storage and outstanding durability and cyclability >> 2000 times (Scheme 2).

## METHODS

### Experimental design of materials and electrodes

A diverse variety of super-hierarchical materials of anodic TO, LSO, LSO.TO and cathodic LMPO compositions were synthesized by the specific synthesis protocols under hydrothermal treatments (see Supporting information S1). A simple carbonization process was carried out to fabricate LSO.TO@nano-C and LMPO@nano-C using a microwave radiation technique, as reported in Supporting information S1.

Formulation of the super-hierarchical LMPO and LMPO@nano-C cathodes was carried out by specific mass-loading of materials into aluminum (Al) foils (i.e. P-electrodes). The TO, LSO, LSO.TO and LSO.TO@nano-C anodes were fabricated using 8 μm-cupper (Cu) foils (i.e. N-electrodes) as platforms. Both super-hierarchical P- and N-electrodes were successfully fabricated under specific synthesis protocols, as reported in Supporting information S1.

### Fabrication sets of half- and full-system LIB-CR2032 coin cell models

Half-cell anodic electrode batteries were fabricated using TO, LSO, LSO.TO and LSO.TO@nano-C architects. The half-cell cathodic electrode batteries were successfully prepared using LMPO or LMPO@nano-C composites. The LSO.TO@nano-C anodic and LMPO@nano-C cathodic electrodes were modulated into full-system LIB-CR2032 coin cell models (see Supporting information S1–S18).

Formation of LSO.TO@nano-C and LMPO@nano-C electrodes enabled formulations of half- and full-scale LIB-CR2032 coin cells. LIB coin cells were formulated in 16 mm circular-shaped Li-foil electrodes (i.e. counter and reference electrodes). A 20-mm circular microporous polymeric membrane separator and 1 M conductive LiPF_6_ solution in a mixture of ethylene carbonate/diethyl carbonate (1:1 v/v) were used for assembly of half- and full-cell LIBs. The active components of cathode and anode materials were incorporated into 10 μm-Al and 8 μm-Cu foil thicknesses. These anisotropic super-hierarchical architectures of cathode and anode electrodes were basically used as working P- and N-electrodes, respectively, as reported in Supporting information S1.

With the aim of improving safety issues and maintaining high LIB performances, robust packing of material components onto the electrode surfaces, in addition to the coin cell design, is crucial. The compact packing process will enhance electrical contact between the multi-reactive centering components/sites, and solid electrode surface/liquid electrolyte interfaces (SEI). A set of experiments was conducted to control the optimal performance of P- and N-electrodes, and coin cells under specific compressing conditions. To design robust LIB models, we used a coin cell crimper of CR20XX series in a clean glove box under Ar gas. Our compressing control was based on a specific mechanical processing protocol, which significantly offers compact circular 2032 coin cells and electrodes (see Supporting information S1–S18).

Overall, fabrication sets of full-system LIB-CR2032 coin cell models were designed for high electrochemical performance (Scheme 2). To investigate the outstanding, large-scale models in fabricated LIB modules, we designed ordered sets of multiple rolls of coin cells, and then packed these up into a collar fashion of 18650-cylindrical LIB modes or stacking layer configurations of pouch LIB types. Aiming to facilitate electrochemical measurements, the LSO.TO@nano-C and LMPO@nano-C stacked pouch LIB-types are generally specified as special models in this study. Rational control of mass loading balance between N- and P-electrodes is crucial for the tradeoff between improvement of high specific energy density and attainment of the intrinsic safety of LIBs. The N/P balancing ratios of LSO.TO@nano-C-anode and LMPO@nano-C-cathode electrodes in pouch LIB models were obtained through multiple building blocks of stacked 5-/6-layers and along 10 sides of both 8 μm-Cu and 10 μm-Al electrodes, respectively (see Supporting information). Our ordered sets of LSO.TO@nano-C and LMPO@nano-C layers designated in pouch LIB-types provide outstanding tradeoff in terms of improved safety with maintenance of high specific energy density and long-life cycles, as required for power-extended EV driving range.

## CONCLUSION

We report on anisotropic alignments of hierarchical LSO.TO@nano-C anode//LMPO@ nano-C cathode architectures for half- and full-system LIB-CR2032 coin cell models. The full-scale LIB coin cell models provide high energy density, superior durability/cyclability, Coulombic efficiency and outstanding rate capability. Along with fabrication of LIB coin cell models, we explored the incentives and keys of anode/cathode architectures with anisotropic surface heterogeneity, multi-component reactive plane sites, dimension scales and vacancies, and composite textures on their outstanding electrochemical performance. The formulation of full-system LIB-CR2032 coin cell models offers considerable insight into storage systems in terms of maximum capacitance and power energy density. Large-scale LIB-ordered set models were designed with use of multiple rolls of coin cells, and then packed-up into a collar fashion of 18650-cylindrical and stacking pouch LIB designs. As a promising avenue for development of long-range EVs, the hierarchal architecture-driven LSO.TO@nano-C//LMPO@nano-C pouch LIB-configurations produce high energy density of 237.6 Wh kg^−1^ [1]. For long-term energy storage, the retention of cycled anode/cathode electrode orientations and dimensions after multiple charge/discharge cycles enables creation of hierarchal LIB models with continuous in-/out-flow rates and fast transport pathways of Li^+^-ions. We show superior durability of LIB models in terms of excellent capacity retention (∼89.8% after long-term cycling period of 2000 cycles) and average Coulombic efficiency of 100% at 1C rate, with future applications in rechargeable batteries.

## Supplementary Material

nwaa017_Supplemental_File

## References

[1] Li S , Wu Q, Zhang D et al. Effects of pulse charging on the performances of lithium-ion batteries. Nano Energy 2019; 56: 555–62.

[2] Huang Y , He Y, Sheng H et al. Li-ion battery material under high pressure: amorphization and enhanced conductivity of Li_4_Ti_5_O_12_. Natl Sci Rev 2019; 6: 239–46. 10.1093/nsr/nwy122 PMC8291545 34691862

[3] Zhang W , Fu Y, Liu W et al. A general approach for fabricating 3D MFe_2_O_4_ (M = Mn, Ni, Cu, Co)/graphitic carbon nitride covalently functionalized nitrogen-doped graphene nanocomposites as advanced anodes for lithium-ion batteries. Nano Energ 2019; 57: 48–56.

[4] Pan Y , Chou S, Liu HK et al. Functional membrane separators for next-generation high-energy rechargeable batteries. Natl Sci Rev 2017; 4: 917–33.

[5] Aravindan V , Gnanaraj J, Lee YS et al. LiMnPO_4_ —a next generation cathode material for lithium-ion batteries. J Mater Chem A 2013; 1: 3518–39.

[6] Choi NS , Chen Z, Freunberger SA et al. Challenges facing lithium batteries and electrical double-layer capacitors. Angew Chem Int Ed 2012; 51: 9994–10024. 10.1002/anie.201201429 22965900

[7] Lee JH , Yoon CH, Hwang JY et al. High-energy-density lithium-ion battery using a carbon-nanotube-Si composite anode and a compositionally graded Li[Ni_0.85_Co_0.05_Mn_0.10_]O_2_ cathode. Energ Environ Sci 2016; 9: 2152–8.

[8] Ji L , Zheng H, Ismach A et al. Graphene/Si multilayer structure anodes for advanced half and full lithium-ion cells. Nano Energy 2012; 1: 164–71.

[9] Chan CK , Peng H, Liu G et al. High-performance lithium battery anodes using silicon nanowires. Nat Nanotechnol 2008; 3: 31–5. 18654447 10.1038/nnano.2007.411

[10] Ji L , Zhang X. Evaluation of Si/carbon composite nanofiber-based insertion anodes for new-generation rechargeable lithium-ion batteries. Energ Environ Sci 2010; 3: 124–9.

[11] Ji L , Lin Z, Alcoutlabi M et al. Recent developments in nanostructured anode materials for rechargeable lithium-ion batteries. Energ Environ Sci 2011; 4: 2682–9.

[12] Traoré S , Traoré DL, Kourouma SY et al. Preparation of silicon from rice husk as renewable energy resource by the use of microwave ashing and acid digestion. Inter J Energ Eng 2018; 8: 25–9.

[13] Li C , Wang T, Liu B et al. Photoelectrochemical CO_2_ reduction to adjustable syngas on grain-boundary-mediated a-Si/TiO_2_/Au photocathodes with low onset potentials. Energ Environ Sci 2019; 4: 10–4.

[14] Sealy C . Lithium-ion batteries charge to the next level. Mater Today 2018; 21: 588–9.

[15] Ryu J , Hong D, Lee HW et al. Practical considerations of Si-based anodes for lithium-ion battery applications. Nano Res 2017; 10: 3970–4002.

[16] Hwa Y , Kim WS, Yu BC et al. Facile synthesis of Si/TiO_2_(anatase) core-shell nanostructured anodes for rechargeable Li-ion batteries. J Electroanal Chem 2014; 712: 202–6.

[17] Loveridge MJ , Lain MJ, Johnson JD et al. Towards high capacity li-ion batteries based on silicon-graphene composite anodes and sub-micron V-doped LiFePO_4_ cathodes. Sci Rep 2016; 6: 37787. 27898104 10.1038/srep37787 PMC5127186

[18] Saravanan K , Balaya P, Reddy MV et al. Morphology controlled synthesis of LiFePO_4_/C nanoplates for Li-ion batteries. Energ Environ Sci 2010; 3: 457–64.

[19] Wang C , Wu H, Chen Z et al. Self-healing chemistry enables the stable operation of silicon microparticle anodes for high-energy lithium-ion batteries. Nat Chem 2013; 5: 1042–8. 24256869 10.1038/nchem.1802

[20] Liu H , Bi Z, Sun XG et al. Mesoporous TiO_2_-B microspheres with superior rate performance for lithium ion batteries. Adv Mater 2011; 23: 3450–4. 21721051 10.1002/adma.201100599

[21] Wang B , Xin H, Li X et al. Mesoporous CNT@TiO_2_-C nanocable with extremely durable high rate capability for lithium-ion battery anodes. Sci Rep 2014; 4: 3729. 24429419 10.1038/srep03729 PMC3893646

[22] Carp O , Huisman CL, Reller A. Photoinduced reactivity of titanium dioxide. Prog Solid State Chem 2004; 32: 33–177.

[23] Ng SH , Wang J, Wexler D et al. Amorphous carbon-coated silicon nanocomposites: a low-temperature synthesis via spray pyrolysis and their application as high-capacity anodes for lithium-ion batteries. J Phys Chem C 2007; 111: 11131–8.

[24] Xiao Q , Zhang Q, Fan Y et al. Soft silicon anodes for lithium ion batteries. Energ Environ Sci 2014; 7: 2261–8.

[25] Teki R , Datta MK, Krishnan R et al. Nanostructured silicon anodes for lithium Ion rechargeable batteries. Small 2009; 5: 2236–42. 19739146 10.1002/smll.200900382

[26] Peled E . The electrochemical behavior of alkali and alkaline earth metals in nonaqueous battery systems-the solid electrolyte interphase model. J Electrochem Soc 1979; 126: 2047–51.

[27] Terranova ML , Orlanducci S, Tamburri E et al. Si/C hybrid nanostructures for Li-ion anodes: an overview. J Power Sources 2014; 246: 167–77.

[28] Shen C , Wang X, Zhang W et al. Direct prototyping of patterned nanoporous carbon: a route from materials to on-chip devices. Sci Rep 2013; 3: 2294. 23887486 10.1038/srep02294 PMC3724177

[29] Wang G , Qi W, Zhang D et al. Rational design of porous TiO_2_@N-doped carbon for high rate lithium ion batteries. Energ Technol 2018; 7: 1800911.

[30] Lee D-H , Lee B-H, Sinha AK et al. Engineering titanium dioxide nanostructures for enhanced lithium-ion storage. J Am Chem Soc 2018; 140: 16676–84. 30418777 10.1021/jacs.8b09487

[31] Yan D , Bai Y, Yu C et al. A novel pineapple-structured Si/TiO_2_ composite as anode material for lithium ion batteries. J Alloys Compd 2014; 609: 86–92.

[32] Liang J , Wang Z, Li Z et al. Fabrication of nanostructured TiO_2_ using a solvothermal reaction for lithium-ion batteries. Nanomater Nanotechnol 2016; 6: 15.

[33] Zhang M , Garcia-Araez N, Hector AL. Understanding and development of olivine LiCoPO_4_ cathode materials for lithium-ion batteries. J Mater Chem A 2018; 6: 14483–517.

[34] Chen D , Wei W, Wang R et al. Facile synthesis of 3D hierarchical foldaway-lantern-like LiMnPO_4_ by nanoplate self-assembly, and electrochemical performance for Li-ion batteries. Dalt Trans 2012; 41: 8822–8. 10.1039/c2dt30630a 22692085

[35] Zhu JN , Li WC, Cheng F et al. Synthesis of LiMnPO_4_/C with superior performance as Li-ion battery cathodes by a two-stage microwave solvothermal process. J Mater Chem A 2015; 3: 13920–5.

[36] Recham N , Oró-Solé J, Djellab K et al. Hydrothermal synthesis, silver decoration and electrochemistry of LiMPO_4_ (M = Fe, Mn, and Co) single crystals. Solid State Ion 2012; 220: 47–52.

[37] Khalifa H , El-Safty SA, Reda A et al. Meso/macroscopically multifunctional surface interfaces, ridges, and vortex-modified anode/cathode cuticles as force-driven modulation of high-energy density of LIB electric vehicles. Sci Rep 2019; 9: 14701. 31605015 10.1038/s41598-019-51345-z PMC6789099

[38] Khalifa H , El-Safty SA, Reda A et al. Theoretical and experimental sets of choice anode/cathode architectonics for high-performance full-scale LIB built-up models. Nano-Micro Lett 2019; 11: 84. 10.1007/s40820-019-0315-8 PMC7770700 34138059

[39] Zhou F , Zhu P, Fu X et al. Comparative study of LiMnPO_4_ cathode materials synthesized by solvothermal methods using different manganese salts. CrystEngComm 2014; 16: 766–74.

[40] Hassen D , Shenashen MA, El-Safty SA et al. Nitrogen-doped carbon-embedded TiO_2_ nanofibers as promising oxygen reduction reaction electrocatalysts. J Power Sources 2016: 330: 292–303.

[41] Shenashen MA , Hassen D, El-Safty SA et al. Axially oriented tubercle vein and X-crossed sheet of N-Co_3_O_4_@C hierarchical mesoarchitectures as potential heterogeneous catalysts for methanol oxidation reaction. Chem Eng J 2017; 313: 83–98.

[42] Wu H , Chan G, Choi JW et al. Stable cycling of double-walled silicon nanotube battery anodes through solid-electrolyte interphase control. Nat Nanotechnol 2012; 7: 310–5. 22447161 10.1038/nnano.2012.35

[43] Khairy M , El-Safty SA. Hemoproteins–nickel foam hybrids as effective supercapacitors. Chem Commun 2014; 50: 1356–8. 10.1039/c3cc48155g 24346228

[44] Akhtar N , Emran M, Shenashen MA et al. Fabrication of photo-electrochemical biosensors for ultrasensitive screening of mono-bioactive molecules: the effect of geometrical structures and crystal surfaces. J Mater Chem B 2017; 5: 7985–96. 32264199 10.1039/c7tb01803g

[45] Khairy M , El-Safty SA. Nanosized rambutan-like nickel oxides as electrochemical sensor and pseudocapacitor. Sensor Actuat B Chem 2014; 193: 644–52.

[46] Hassen D , Shenashen MA, El-Safty AR et al. Anisotropic N-Graphene-diffused Co_3_O_4_ nanocrystals with dense upper-zone top-on-plane exposure facets as effective ORR electrocatalysts. Sci Rep 2018; 8: 3740. 29487302 10.1038/s41598-018-21878-w PMC5829235

[47] Huang LW , Elkedim O, Nowak M et al. Synergistic effects of multiwalled carbon nanotubes and Al on the electrochemical hydrogen storage properties of Mg_2_Ni-type alloy prepared by mechanical alloying. Int J Hydrogen Energy 2012; 37: 1538–45.

[48] Hassen D , El-Safty SA, Tsuchiya K et al. Longitudinal hierarchy Co_3_O_4_ mesocrystals with high-dense exposure facets and anisotropic interfaces for direct-ethanol fuel cells. Sci Rep 2016; 6: 24330. 27075551 10.1038/srep24330 PMC4830963

[49] Khairy M , El-Safty SA. Promising supercapacitor electrodes based immobilization of proteins onto macroporous Ni foam materials. J Energy Chem 2015; 24: 31–8.

[50] Yang S , Feng X, Zhi L et al. Nanographene-constructed hollow carbon spheres and their favorable electroactivity with respect to lithium storage. Adv Mater 2010; 22: 838–42. 20217794 10.1002/adma.200902795

[51] Khairy M , El-Safty SA. Mesoporous NiO nanoarchitectures for electrochemical energy storage: influence of size, porosity, and morphology. RSC Adv 2013; 3: 23801–9.

