# Supplementary material for: Anisotropic alignments of hierarchical Li2SiO3/TiO2 @nano-C anode//LiMnPO4@nano-C cathode architectures for full-cell lithium-ion battery
Source: Natl Sci Rev. 2020 Feb 11;7(5):863–80. doi: 10.1093/nsr/nwaa017 (PMC8289010; doi:10.1093/nsr/nwaa017)
Supplement: nwaa017_Supplemental_File [file nwaa017_supplemental_file.docx]

**Anisotropic alignments of hierarchical Li_2_SiO_3_/TiO_2_ @nano-C anode// LiMnPO_4_@nano-C cathode architectures for full-cell lithium-ion battery**

**H. Khalifa, S.A. El-Safty (🖂), A. Reda, M.A. Shenashen, A. I. Eid**

*Research Center for Functional Materials, National Institute for Materials Science (NIMS), Sengen 1-2-1, Tsukuba, Ibaraki 305-0047, JAPAN.*

*Corresponding Author Information: Sherif A. El-Safty*

*E-mail:* [*sherif.elsafty@nims.go.jp*](mailto:sherif.elsafty@nims.go.jp)*;*

*Webpage:* [*https://samurai.nims.go.jp/profiles/sherif_elsafty*](https://samurai.nims.go.jp/profiles/sherif_elsafty)

**S1. Experimental design**

**S1-A: Materials and experimental sections**

High grade chemicals are used without further purification. Lithium hydroxide monohydrate (LiOH.H_2_O), ascorbic acid (C_6_H_8_O_6_), hydrogen peroxide (H_2_O_2_) and hexadecyltrimethylammonium bromide (CTAB-C_19_H_42_BrN) are produced by Sigma–Aldrich Company, Ltd., USA. Titanium (IV)-oxysulfate (titanoxysulfat-TiO(SO_4_).xH_2_O), ethylene glycol (C_2_H_6_O_2_) and ammonia solution (NH_4_OH) from Nacali Tesque Company, Ltd., Kyoto, Japan. Tetramethyl orthosilicate (SiC_4_H_12_O_4_), potassium permanganate (KMnO_4_) and phosphoric acid (H_3_PO_4_) are purchased from Tokyo Chemical Industry (TCI) Company, Ltd., Tokyo, Japan. Lithium chloride (LiCl) and salicylic acid (C_7_H_6_O_3_) are produced from Wako Company, Ltd., Osaka, Japan. The anisotropic architectures of TiO_2_, Li_2_SiO_3_, Li_2_SiO_3_.TiO_2_ and LiMnPO_4_ compositions are synthesized by the hydrothermal treatment method. In turn, the carbonization of Li_2_SiO_3_.TiO_2_ and LiMnPO_4_ are carried out to fabricate by microwave radiation technique.

**S1-B. Fabrication of hierarchal architects**

**Fabrication of TO, LSO, and LSO.TO anode architects**

Four steps of time-dependent heterogeneous particle-to-particle diffusion are used to fabricate LSO.TO@nano-C anisotropic super-hierarchical architectures.

First, TO is formed as follows: a 159.93 mg of titanoxysulfate (TiO(SO_4_).xH_2_O) is dissolved in a mixture of 2 M salicylic acid (C_7_H_6_O_3_), 30 mL of ethanol, 30 mL of Milli-Q water, and 4 mL of H_2_O_2_ solution and then stirred for 1 h. Subsequently, 5 mL of NH_4_OH (ca. 4% in ethanol, ca. 2.0 mol/L) is added drop-wise at 0.5 mL/min under vigorous and continuous stirring for 30 min.

Second, LSO is formed as follows: a 15 mL of tetramethylorthosilicate solution and ethylene glycol (EG) are added dropwise into a 20 mL solution mixture of LiOH/H_2_O, 15 mL of ethanol, and 10 mL of EG at 0.5 mL/min. The sample composition solution (Si: Li=1:1 in molar ratio) is stirred vigorously.

Third, LSO.TO is formed as follows. The as-made LSO and TO mixtures with molar ratio (Si:Ti=1:1) is controlled by using the original composition sample of as-made TO and LSO. The TiO_2_/Li_2_SiO_3_ sample is transferred into 100 mL Teflon-lined stainless-steel autoclaves maintained at 170 °C for 12 h and cooled to room temperature. The resulting solid products are centrifuged, repeatedly washed with Milli-Q-water and absolute ethanol, and dried overnight at 60 °C under vacuum. The resulting solid sample is calcined in a muffle furnace under Ar at 600 °C for 2 h to form LSO.TO white powder. The white TO and LSO solid products are fabricated by the same synthesis route as that of LSO. Sample fabrication is performed in accordance with the third fabrication step of hydrothermal and high-temperature treatment protocol.

**Fabrication of LMPO formulated cathode architects**

LiMnPO_4_ is fabricated by using typical time-dependent heterogeneous-particle diffusion. a mixture of 10 mL of LiCl, 40 mL of Milli-Q water, and 10 mL of EG is added dropwise (0.5 mL/min) to another mixture of phosphoric acid, potassium permanganate, and ascorbic acid with specific Li: Mn: P: ascorbic acid molar ratio of 3:1:1:0.5, respectively. The mixture is transferred to 100 mL Teflon-lined stainless-steel autoclaves and treated at 170 °C for 12 h. The resulting solid product is centrifuged, dried overnight at 60 °C, and calcined under Ar at 600 °C for 2 h to form a light-gray LiMnPO_4_ powder.

**Fabrication of LSO.TO@nano-C/LMPO@nano-C formulated anode/cathode architects**

A microwave-assisted approach is used to well-defined dressing of both Li_2_SiO_3_/TiO_2_-anatase and olivine LiMnPO_4_ multilayer-stacked bowtie antenna by nano-carbon-bumps. In typical dressing protocol, glucose (5 wt%) is added to the as-prepared LSO.TO and LMPO samples. The glucose-dressed LSO.TO and LMPO are ground, spread into Milli-Q water/ethanol, and ultrasonicated for 15 min. The LSO.TO/glucose and LMPO/glucose mixtures are transferred to autoclaves and stirred for 30 min under microwave irradiation at 80 °Ϲ. The color of both mixtures finally turned black. LSO.TO@nano-C and LMPO@nano-C precipitates are collected by centrifugation, washed with Milli-Q water and ethanol, and dried overnight at 55 °Ϲ. The resulting samples are calcined in Ar atmosphere at 350 °C for 0.5 h and set at 600 °C for 2 h with a heating rate of 5 °C/min. The final products for Li_2_SiO_3_.TiO_2_@nano-C and LiMnPO_4_@nano-C powders are labeled as LSO.TO@nano-C and LMPO@nano-C, respectively. Note that: The formulations of N-anode and P-cathode electrodes using these fabricated [LSO.TO@nano-C](mailto:LSO.TO@%20C) and LMPO@nano-C materials in specific synthesis protocol, respectively (see S1-E). Both N-anode and P-cathode electrodes were designated in CR2032 coin cells and used in half- and full-cell LIBs, are controlled under specific protocols, as reported in Supporting Information S1 (Figure S1).

**S1- C- Characterizations of LMPO@nano-C cathode and LSO.TO@nano-C anode structures**

X-ray diffraction (XRD) is performed to investigate the crystal structures of the LMPO and LMPO@nano-C cathodes- and TO, LSO, LSO.TO, and LSO.TO@nano-C anodes-based architects using a 18 kW diffractometer (Bruker D8 Advance X-ray diffractometer) at scan rate of 10°/min with CuKα-X-radiation (λ = 1.54178 Å) at 30 kV and 10 mA. Field emission-type scanning electron microscope (FESEM) associated with energy-dispersive X-ray unit (EDX) are used to investigate the morphological components, compositions and structural shapes, and elemental distribution mapping along LMPO@nano-C cathode and LSO.TO@nano-C anode architects. The LMPO and LMPO@nano-C cathodes- and TO, LSO, LSO.TO, and LSO.TO@nano-C anodes-based morphological architects are investigated by FE-SEM (Jeol JSM-Model 7000F, JEOL Ltd) at 20 kV. The FE-SEM is equipped with a Schottky (thermal type) field emission electron gun. Analysis material is fixed onto the FE-SEM stage using carbon tape before insertion into the FE-SEM chamber. The ion sputter (Hitachi E-1030) is used to deposit thin-layered Pt films on electrodes at 25 °C. To study the atomic-scale arrangements and structural crystal and surface orientation of LMPO and LMPO@nano-C cathodes- and TO, LSO, LSO.TO, and LSO.TO@nano-C anodes-based architects, high-resolution transmission electron microscopy (HRTEM) images, electron diffraction (ED), scanning transmission electron microscopy (STEM) are performed at atomic-level imaging, structural and chemical analysis field emission-type TEM (JEM-ARM200F). The accelerating voltage can be adjusted to 80, 120 or 200 kV, depending on the specimen material and the purpose of observation. Energy-dispersive X-ray spectroscopy (STEM-EDS) is carried out with high-resolution elemental mapping for LMPO and LMPO@nano-C cathodes- and TO, LSO, LSO.TO, and LSO.TO@nano-C anodes-based architects to investigate the chemical contents of the nanostructures by using a 200 kV TEM (JEOL 2100F, JEOL Ltd) field emission-type transmission electron gun microscope. The LMPO and LMPO@nano-C cathodes- and TO, LSO, LSO.TO, and LSO.TO@nano-C anodes-based samples are dispersed in ethanol solution using an ultrasonic radiation, and then dropped on a copper mesh and vacuum dried for 20 min prior to inserting the samples into the HR-TEM and STEM-EDS columns. The surface textural and parameters, such as pore structure distribution and surface area, of LMPO and LMPO@nano-C cathodes- and TO, LSO, LSO.TO, and LSO.TO@nano-C anodes-based architects are estimated by N_2_ adsorption–desorption isotherms at 77 K using a BELSORP36 analyzer (JP. BEL Co., Ltd.). The LMPO@nano-C cathode and LSO.TO@nano-C anode structures are thermally treated at 200 °C for at least 6 h under N_2_ atmosphere. The specific surface area (S_BET_) is calculated by using the Brunauer–Emmett–Teller (BET) method with multipoint adsorption data from the linear section of the N_2_ adsorption isotherm. The pore size distribution is determined by using nonlocal density function theory (NLDFT). The weight components, amount ratios of LMPO and LMPO@nano-C cathodes- and TO, LSO, LSO.TO, and LSO.TO@nano-C anodes-based architect composites are determined from the weight loss curve measured under simulated air atmosphere on a TG/DSC instrument TG/DSC-60 (Shimadzu, Japan) with a heating rate of 10 ^0^C min^-1^. These TG/DSC analyses were used to approve the N- and P-electrode materials stability under high-temperature heating. X-ray photoelectron spectroscopy (XPS) analysis (0-1400eV) of LMPO and LMPO@nano-C cathodes- and TO, LSO, LSO.TO, and LSO.TO@nano-C anodes-based architects is conducted on a PHI Quantera SXM (ULVAC-PHI) instrument (Perkin–Elmer Co., USA) equipped with Al K_α_ radiation as an X-ray source for excitation (1.5 mm × 0.1 mm, 15 kV, 50 W) under a pressure of 4×10^−8^ Pa. To study the chemical structure analyses, Raman spectroscopy (HR Micro Raman spectrometer, Horiba, Jobin Yvon) is conducted for LMPO and LMPO@nano-C cathodes- and TO, LSO, LSO.TO, and LSO.TO@nano-C anodes-based architects. Raman studies are by using an Ar ion laser at 633 nm. A charge coupled device (CCD) camera detection system and the LabSpec-3.01C software package are used for data acquisition and analysis, respectively. To ensure the accuracy and precision of the Raman spectra, 10 scans of 5s from (500-2000 cm^−1^) are recorded. The chemical bonding and interaction, and structural compositions of the LMPO and LMPO@nano-C cathodes- and TO, LSO, LSO.TO, and LSO.TO@nano-C anodes-based architects are analyzed by Fourier transform infrared spectroscopy (ATR-FTIR, Spectrum 100, Perkin-Elmer, Inc., USA).

**S1- D- Materials used for electrochemical electrode fabrication and its half- and full-scale LIBs**

All consumed chemicals have high analytical grade and used without further purification. Lithium hexafluoro-phosphate LiPF_6_, carbon black and polyvinylidene fluoride (PVDF) are supplied from Sigma–Aldrich Company, Ltd., USA. N-methyl-2-pyrrolidone (NMP) is produced from Tokyo Chemical Industry (TCI) Company, Ltd., Tokyo, Japan. Electrochemical measurement of Li-ion intercalation is performed by using CR2032 coin-cells that accumulated in a glovebox under argon gas, Figure S1. Half LIB-coin-cells are consisted of (i) working electrode that incorporated with mass loading of active architect materials (such as a family of [LSO.TO@nano-C-anode](mailto:LSO.TO@C-anode) or LMPO@nano-C cathode composition), and (ii) the lithium foil applied as reference and counter electrodes, respectively. Anode half-cell batteries are fabricated by using TiO_2_ (TO), Li_2_SiO_3_ (LSO), Li_2_SiO_3_.TiO_2_ (LSO.TO) or Li_2_SiO_3_.TiO_2_@nano-C (LSO.TO@nano-C) as working electrodes. The cathode half-cell batteries are prepared by using of LiMnPO_4_ (LMPO) or LiMnPO_4_@nano-C (LMPO@nano-C) composites as cathodic working electrodes. Moreover, the full-scale [LSO.TO@nano-C](mailto:LSO.TO@nano-C) (anode)//LMPO@nano-C (cathode) LIB- CR2032 coin cell is also successfully fabricated for electrochemical measurements by integration of anodic LSO.TO@nano-C and cathodic LMPO@nano-C electrodes in same cell.

The working electrodes are prepared by mixing specific amounts of active architect materials of LSO.TO@nano-C anode composite and LMPO@nano-C cathode materials with carbon black. To this anodic or cathodic mixture, a binder such as polyvinylidene fluoride (PVDF) is added. The overall weight ratio of active materials (anode or cathode: carbon black: PVDF-binder) is of 75: 15: 10, respectively. The overall active material compositions is dissolved in N-methyl-2-pyrrolidone (NMP) under stirring-assisted method for 1 h. Simple casting process of the resultant slurries onto aluminum foil (10 µm thickness and 16 mm diameter) surfaces for LMPO or LMPO@nano-C cathode compositions and onto copper foil (8 µm thickness and 16 mm diameter) for TO or LSO, or LSO.TO or LSO.TO@nano-C anode compositions is successfully achieved. The drying of working electrodes is occurred in a vacuum-oven at 80 ^o^C for 12 h.

In the modulation of LIBs, we used a 20-mm-microporous polymeric membrane separator of Celgard 2400^TM^ membrane that produced from Hoechst Celanese Corporation, Charlotte, North Carolina, USA to enhance the diffusivity, mass-transports and in-/out-flow rates of electron/Li^+^ ion movements during lithiation/delithiation cycling. Furthermore, the liquid electrolyte mixtures used in these hierarchal CR2032 coin-cell LIBs are highly conductive compounds that are homogenously composed of LiPF_6_ (1 M) and a solution mixture (v/v of 1:1) of ethylene carbonate and diethyl carbonate, respectively. The prepared coin-cell batteries are left for 24 hours prior to the electrochemical measurements.

**S1-E-Electrochemical control measurements**

Galvanostatic charging/discharging characteristics are measured by using multi-channel battery systems of LAND CT2001A, Wuhan, China. Cyclic voltammogram (CV) measurements of the cells are tested by using (CHI 660c electrochemical workstation). The EIS (electrochemical impedance spectroscopy) is performed by using Zennium/ZAHNER-Elektrik GmbH & CoKG, which controlled by Thales Z-3.0 software at a frequency range of 0.1 Hz–1 MHz. Overall electrochemical patterns are investigated at 25 ^o^C.

**
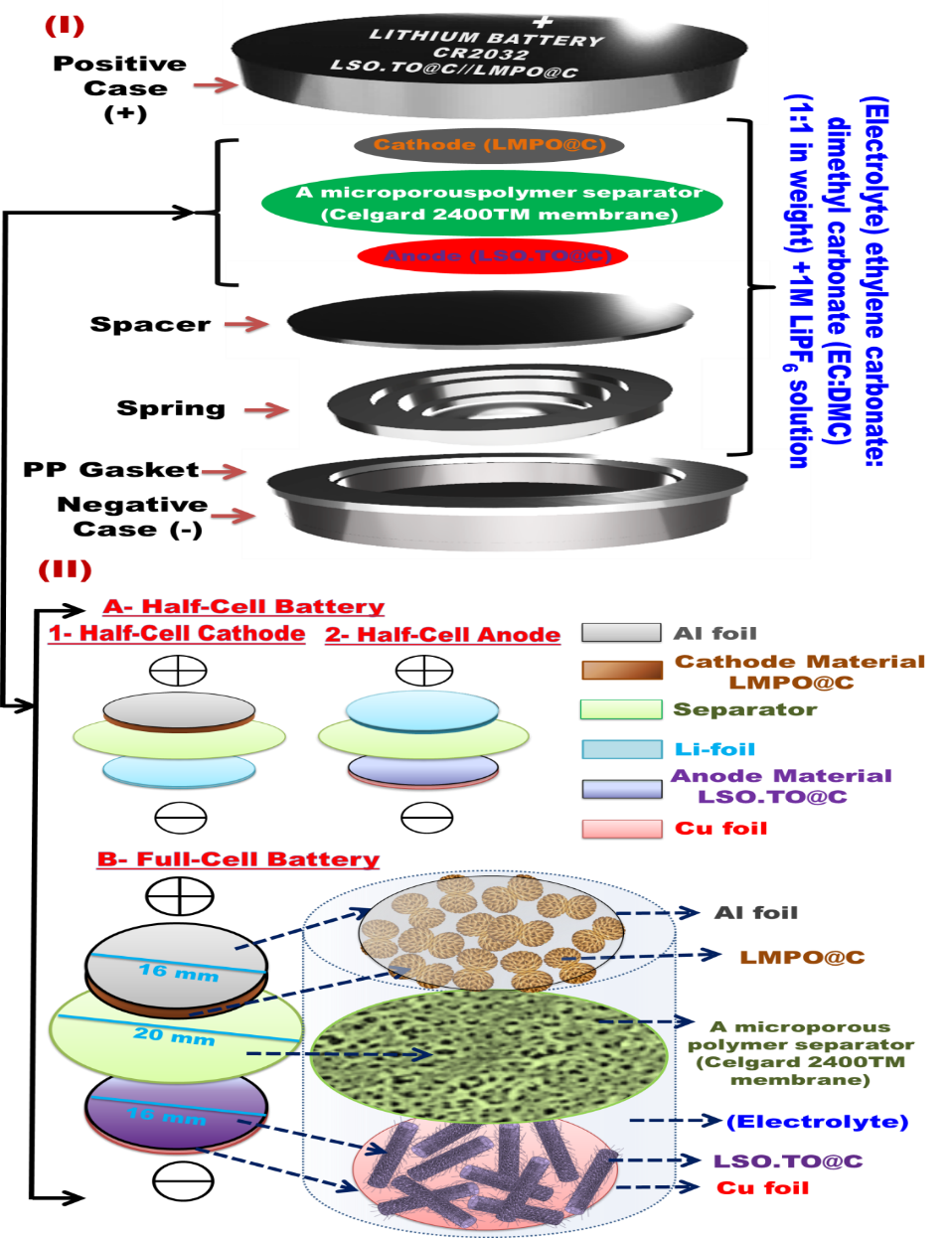
**

**Fig. S1. (I)** Schematic assembly and mechanical control of LIB design based circular CR2032-type coin cells. **(II)** Schematic design of formulation of LSO.TO@nano-C (anode) and LMPO@nano-C (cathode) electrodes designated along the Cu- and Al-foil working electrodes, respectively. The specific formation and collection of the CR2032-type coin cell used in half- and full-scale LIBs.

**S2. Morphological structures of LSO and TO hierarchy anodes**

The topographic morphologies and anisotropic architectures of (i) anodic TiO_2_, Li_2_SiO_3_, Li_2_SiO_3_.TiO_2_ and [Li_2_SiO_3_.TiO_2_@nano-C](mailto:Li2SiO3.TiO2@C), (ii) cathodic LiMnPO_4,_ LiMnPO_4_@nano-C compositions, and (iii) their N- and P-electrodes are characterized by FE-SEM (field emission scanning electron microscope), HRTEM (high-resolution transmission electron microscopy images, (ED) electron diffraction, and (STEM) scanning transmission electron microscopy, respectively (Figure 1 and S2).

The morphologies of parent TO and LSO samples are investigated by using FE-SEM, as shown in (Figure S2). Figure S2-A(a-b) indicates the formation of anatase (TO) with spherically irregular, sizable cotton-like NP morphology. These TO nanoparticles (NPs) aggregated irregularly and formed fenella-elms structure, see inset of Fig.S2. (A-b). The fabrication of groove-space along the nano-hairy/cotton like structure offers pocket accommodation for facile diffusion of the electrons/Li^+^ ions. Figure S2-B(a-b) shows the calcined LSO core-pole rods as platform carriers, leading to a large mass loading of immobilization of reactive component sites along the interior/exterior caves and grooves of LSO rod-like architecture. These architectural designs of both cathode/anode electrodes provide multi-gates for fast diffusion of Li^+^ ions even after multiple lithiation/delithiation processes. The microscopic image (Figure S2 (Ac-d)) shows TO hairy/cotton-shaped particles with a mass of voids and ridges. The TO lattice fringes are observed with the width of 3.495±5 Å, corresponding to the [101] plane. HR-TEM shows that the nano-cotton shape-TiO_2_ samples are highly crystalline-anatase TiO_2_ (JCPDS 21-1272) structure without mixed phases such as rutile and brookite structures. The microscopic HR-TEM image shows that the core-pole rods Li_2_SiO_3_ (LSO) are formed with ~ 0.8µm length and in diameter 75 nm, see Fig.S2.B(c-d). Figure S2(B-e) shows the ED pattern of pure Li_2_SiO_3_ (LSO) rod-core with main dominates along [111] plane, corresponding to the present of Si-composite. It is important to note that the mechanical-chemical energy injected via stirring and hydrothermal processes is sufficient to mix the 0.8µm core rods length of LSO and TO cotton-hairy structure to synthesize a large LSO.TO core-shell rod skewers (1~1.1µm) with LSO core and TO hairy/feathery needles. In addition, HR-TEM and ED images showed well-developed LSO.TO fringes with pure crystalline phases with dominates diffraction [111]/[101] planes, referring to pure Li_2_SiO_3_ (LSO) rod-core and anatase TO shell (Figure S2Ac-d).


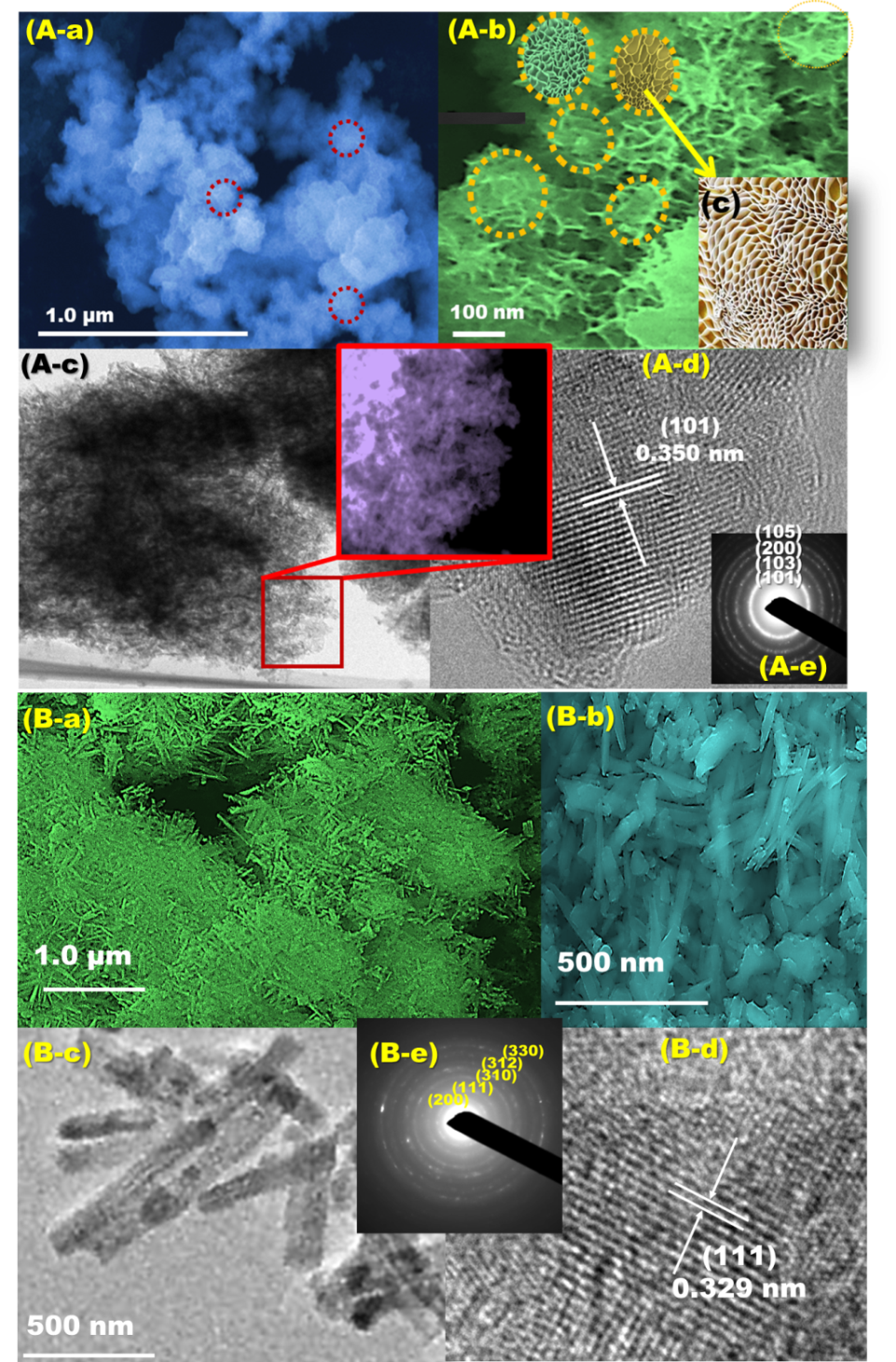


**Fig. S2.** **(A-a, A-b)** Low and high magnification images of FE-SEM microscopes of macro/mesoporous cages and caves of TO cotton-fenella elms-like structure. **(A-c)** HR-TEM image, **(A-c-inset)** high magnification image, **(A-d)** lattice pattern and **(A-e)** ED pattern for TO-anatase architects. **(B-a)** Low and **(B-b)** high magnification of SEM images of LSO nanorods-like structure. **(B-c)** HR-TEM image, **(B-d)** Lattice pattern and **(B-e)** ED pattern for LSO materials.

**S3-1. Crystal Structure formation of TO and LSO hierarchy**

Structural properties of prepared TO and LSO are evaluated by X-ray diffraction (XRD) characterizations Fig. S3(A) and (B); respectively. The crystal lattices of TO are calculated by using refinement protocol and found to be a= 3.789 Å, b= 3.789 Å and c = 9.489 Å. Similarly, all the diffraction peaks of Fig. S3(B) can be assigned to the pure phase of lithium meta-silicate Li_2_SiO_3_ (LSO) [space group of Cmc2_1_, JCPDS 15-0519] without any mixed phases like lithium di-silicate Li_2_Si_2_O_5_.

1. The observed XRD reflections of anatase TO cotton-like NPs can be seen in Fig.S3(A) and indexed according to I41/amd (space group)-tetragonal structure. It is evident from XRD-pattern that the formation of particularly anatase pure structure for -TO material (JCPDS 21-1272) is formed, as evidenced from no diffraction peaks assigned the formation of impurity traces like rutile and brookite structures.
2. The architectural TiO_2_-anatase (TO) is suggested to be used as an excellent anode material owing to its great merits in safety performance, cheap, non-toxic, pure-in natures, eco-friendly, low polarization and good reversible capacity. These features are dependent of the crystal structure transition from I41/amd (space group)-tetragonal TiO_2_ to Imma (space group)-orthorhombic lithium-rich Li_0.5_TiO_2_ geometrics. This phase transition is key function to buildup efficient LIBs based anodic electrodes.
3. Similarly, XRD pattern of LSO core-pole-like NRs is illustrated in Fig.S3(B). As shown in Fig.S3 (B), the peaks can be assigned to the pure-phase of lithium meta-silicate Li_2_SiO_3_ (LSO) [space group of Cmc2_1_, JCPDS 15-0519] without any mixed phases like lithium di-silicate Li_2_Si_2_O_5_ structures.
4. Among several Si composites, the crystal structure surfaces of lithium meta-silicate Li_2_SiO_3_ (LSO) composites show structure stability, and good conductivity, providing novel and typical Li^+^-ion diffusion tunnels during multiple cycles (Figure S3B).
5. These TO and LSO architectures offer multi-directional accommodation sites for facile diffusion of the electrons/Li^+^ ions during lithiation/delithiatin processes.

**
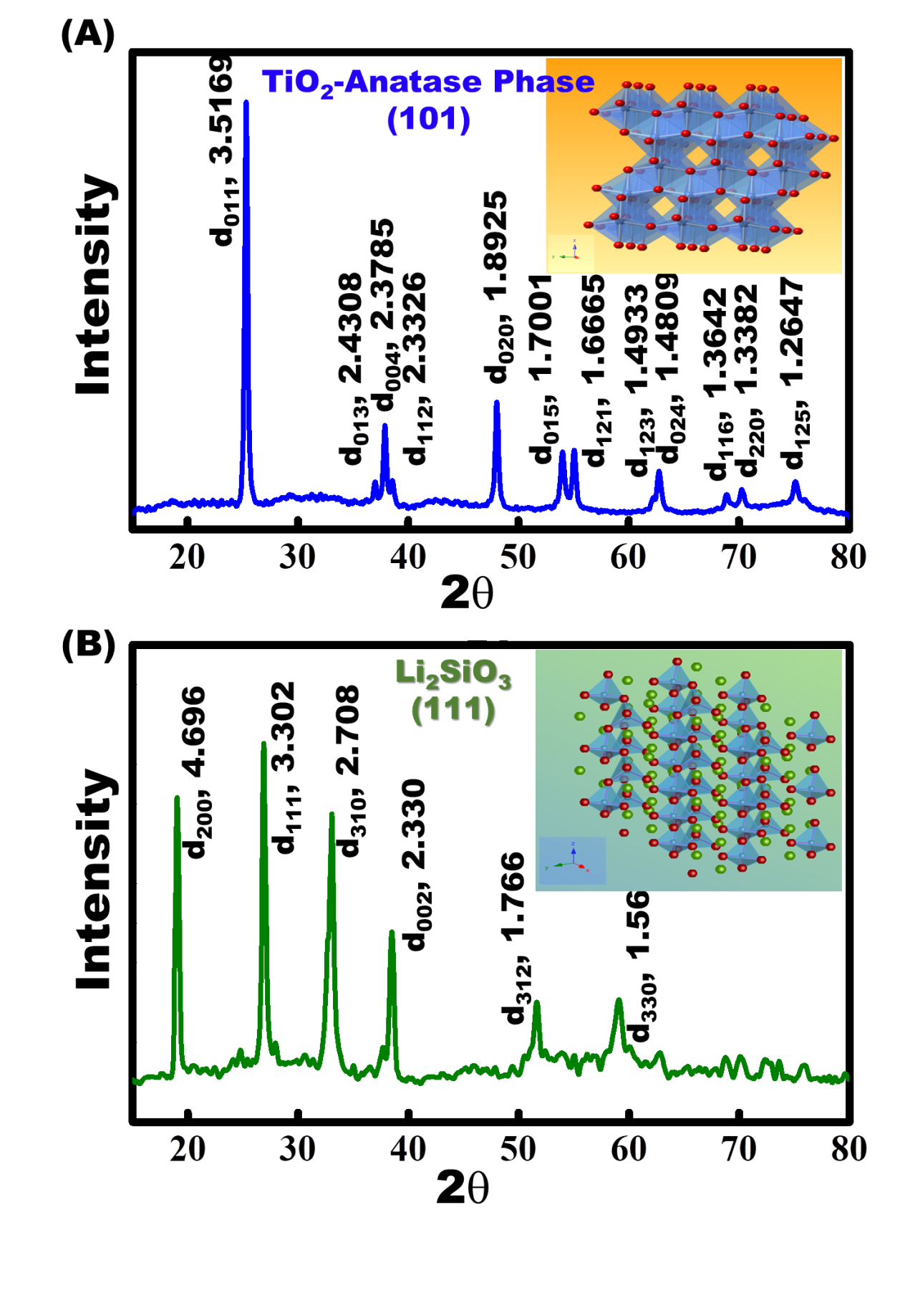
**

**Fig. S3.** **(A)** X-ray diffraction patterns of the as-synthesized TO-anatase structure, **(A-inset)** crystal structure of (101) plane of anatase structure TO. **(B)** X-ray diffraction patterns of the as-synthesized LSO **(B-inset)** crystal structure of (111) plane of LSO composite.

**S3-2. Crystal Structure formation of cathode/anode super-hierarchical architecture crystals**

Figure 1E shows the X-ray diffraction (XRD) profiles of the anisotropic super-hierarchical architectures of anodic TiO_2_, Li_2_SiO_3_, Li_2_SiO_3_.TiO_2_ and [Li_2_SiO_3_.TiO_2_@nano-C](mailto:Li2SiO3.TiO2@C), and cathodic LiMnPO_4,_ LiMnPO_4_@nano-C compositions and their N- and P-electrodes. The significant peaks and positions of the diffraction patterns of LSO.TO and LSO.TO@nano-C composites do not change as compared with those of the parent LSO and TO (Figure S3), indicating the retention of the crystal structures of LSO and TO within the formation of LSO.TO@nano-C composites^39^. Figure 1-II(E) represents the XRD patterns of atomic-scale crystals of LMPO and LMPO@nano-C composites. All diffraction peaks in the patterns can be attributed to the pure orthorhombic olivine LMPO structure with a space group of Pnma (a = 10.4881 Å, b = 6.0680 Å, and c = 4.7508 Å, unit volume = 303.022 Å^3^) in agreement with JCPDS card No. 33-0803^40^. The XRD pattern of LiMnPO_4_ indicates that no formulated composites such as Li_3_PO_4_ and Li_4_P_2_O_7_ can be associated with orthorhombic LMPO single-crystal phase, as evidenced from the EDS energy-dispersive X-ray spectroscopy patterns (Figure 1D-II). Figure 1E shows that the nano-carbon-coated samples LSO.TO@nano-C and LMPO@nano-C decrease the intensity of the peaks of XRD patterns. The diffraction peaks become broader than that patterns obtained for pristine LSO.TO and LMPO samples; however, no changes in the 2-thea position are distinct. Thus, the crystal orientation does not change after forming the LSO.TO and LMPO anode/cathode architectures, and after dressing their surfaces by nano-carbon-bumps.

**S4. Composition distribution of the composite contents along morphological hierarchy**

The STEM-EDS (energy-dispersive X-ray spectroscopy) is applied for determination of high-resolution elemental mappings for TO, LSO, LSO.TO@nano-C and LMPO@nano-C elecrodes, as shown in Fig. S4(A), S4(B), Fig.a.1(C-a) and (C-b), respectively. It is evident that anatase TO cotton-like NPs contain 58.67% (O) and 41.33% (Ti), Fig. S4(A). Also, the LSO core-pole-like NRs composites are 70.40% (O) and 29.60% (Si) contents. However, no identification of Li content is presented, Fig. S4(B). The EDS results indicate the purity of TO and LSO samples with no evidence of other contents of elements.


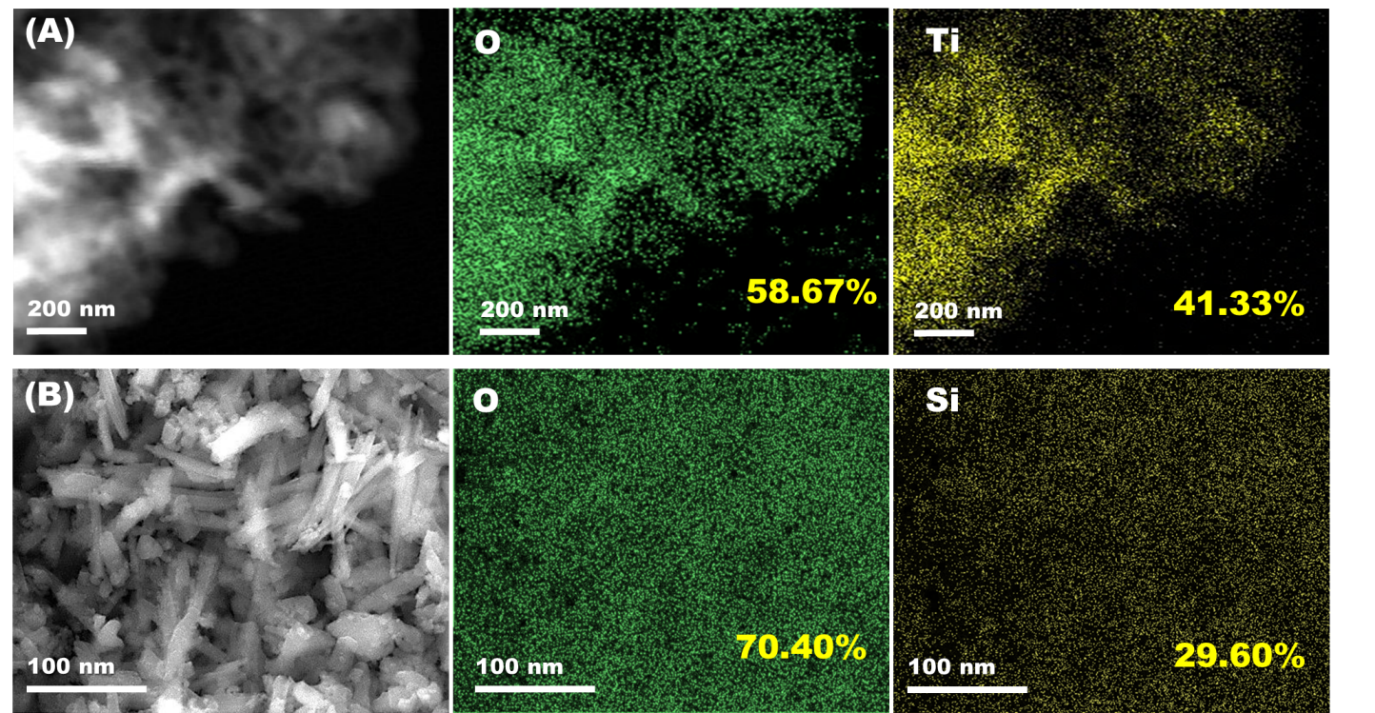


**Fig. S4** FE-SEM images (A& B) and EDX-elemental mapping of the morphologies, composition domains, elemental distribution mapping along architectural designs of **(A)** TO and **(B)** LSO composites.

**S5. Surface parameters of** **anisotropic architectures**

The surface properties of architectural design of anodic TiO_2_, Li_2_SiO_3_, Li_2_SiO_3_.TiO_2_ and [Li_2_SiO_3_.TiO_2_@nano-C](mailto:Li2SiO3.TiO2@C), and cathodic LiMnPO_4,_ LiMnPO_4_@nano-C compositions are determined. The surface parameters involving the surface area and pore size distribution, of N- and P-electrodes are calculated by nitrogen adsorption–desorption isotherms at 77 K.

The specific S_BET_ (surface areas) of anode/cathode architects are estimated by using Brunauer–Emmett–Teller (S_BET_) method. In addition, the pore size distribution (dp/nm) curves are determined by using non-linear density functional theory (NLDFT). N_2_ isothermal shapes enabled the investigation of the interior/exterior shapes of pores, voids and spaces associated with micro-, meso-, and macro-porous architectures of TO, LSO, [LSO.TO@nano-C](mailto:LSO.TO@C) and LMPO@nano-C samples.

Figure S5(A) shows IV-type isotherm with H_2_-hysteresis loop of LMPO and LMPO@nano-C cathodes- and TO, LSO, LSO.TO, and LSO.TO@nano-C anodes-based architects, confirming the formation of dominant mesoporous structure. Figure S5(B) shows the curves of the sizable pores of our tested architects. The Brunauer-Emmett-Teller (S_BET_) surface area values are (106.0,170.8,297.0,368.9,16.72 and 29.9 m^2^g^−1^) and pore size diameters are (24.79,12.08,12.11,11.66,51.32 and 50.56 nm) for (TO, LSO, LSO.TO, LSO.TO@nano-C, LMPO and LMPO@nano-C) materials respectively. The high surface area of nano-carbon coated LSO.TO@nano-C, and LMPO@nano-C samples is evident compared with those free-carbon coated materials. These high surface areas of LSO.TO@nano-C anode /LMPO@nano-C cathode architectures allow large exposure surface potential at solid-electrolyte interface, providing a remarkable tendency to a giant diffusion of electrons/ions dominance. The high surface coverage would enhance the diffusion mobility along the electrode’s active material surfaces during charge/discharge process.

**
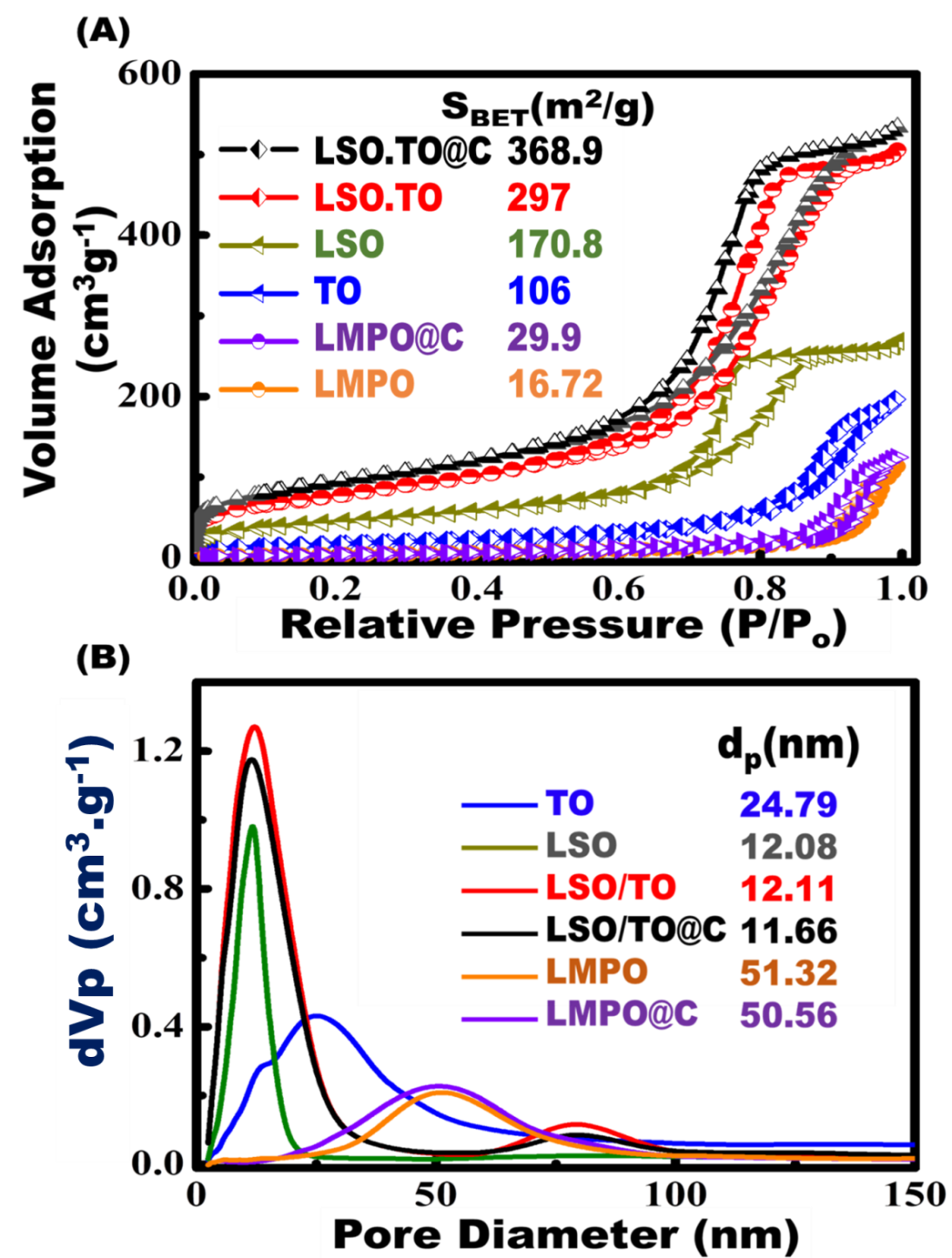
**

**Fig. S5** N_2_-adsorption isotherms data at 77.4 K. (A) N_2_ adsorption isotherm and (B) pore size distribution curves of TO, LSO, LSO.TO, LSO.TO@nano-C, LMPO and LMPO@nano-C.

**S6. Thermal stability of anode and cathode architects**

TG/DSC measurements are performed to investigate the structure stability of anisotropic super-hierarchical architectures of anodic TiO_2_, Li_2_SiO_3_, Li_2_SiO_3_.TiO_2_ and [Li_2_SiO_3_.TiO_2_@nano-C](mailto:Li2SiO3.TiO2@C), cathodic LiMnPO_4,_ LiMnPO_4_@nano-C compositions and their N- and P-electrodes under temperature-treatment >> 800 ^o^C using TG-60 (Shimadzu, Japan) instrument with a heating-rate of 10 ^0^C min^-1^.

**
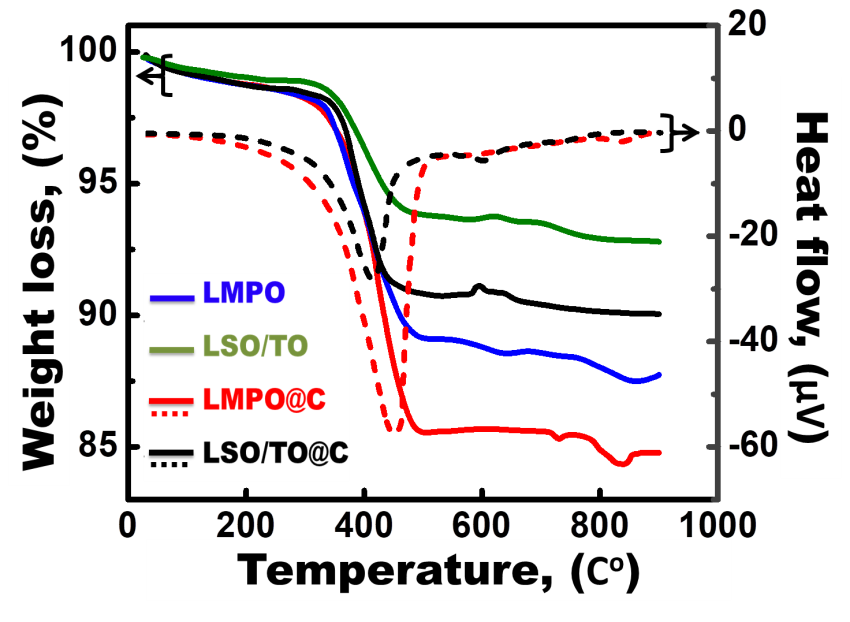
**

**Fig. S6** TG and DSC profiles of the LSO.TO, LSO.TO@nano-C, LMPO and LMPO@nano-C samples

Thermal stability and loss in weight contents of  LMPO and LMPO@nano-C cathodes- and LSO.TO, and LSO.TO@nano-C anodes-based architects are clearly observed by thermogravimetric (TG) method that is analyzed under a continuous-flow of air and with a heating rate of 5 °C·min^−1^, Fig. S6. The TG and DSC profiles exhibit the thermal features of anodic LSO.TO, and LSO.TO /C, and cathodic LMPO and LMPO@nano-C composites. TG/DSC profile showed similar behavior with all LMPO and LMPO@nano-C cathodes- and LSO.TO, and LSO.TO@nano-C anodes-based architects. Three discrete regions of weight loss are described the structural changes under heat treatment. The first insignificant weight-loss region below 350^o^C, is basically produced by releasing of absorbed water and chemisorbed crystal water (desorption of water) from LMPO and LMPO@nano-C cathodes- and LSO.TO, and LSO.TO@nano-C anodes-based architects. The second weight-loss region from 350^o^C to 550^o^C is due to the change of weight-loss of LMPO and LMPO@nano-C cathodes- and LSO.TO, and LSO.TO@nano-C anodes-based architects. It is also suggested that nano-carbon coated LMPO@nano-C and LSO.TO@nano-C samples is very stable because of the pyrolysis of organic compounds. The third weight loss or heat transfer region above 600^o^C is evident of an insignificant change of weight loss or heat transfer that usually occurred when the temperature is further increased to 900^o^C. For temperature treatment > 600^o^C, an exothermic peak is not discovered in DSC curves and the mass of the samples is not changed. This finding indicates that the LMPO and LMPO@nano-C cathodes- and LSO.TO, and LSO.TO@nano-C anodes-based architects are thermodynamically stable in atomic-scale frameworks.

**S7. Functional surface formulation with the anode/cathode composites**

The chemical compositions and atomic-scale framework structure of the TO, LSO, LSO.TO, and LSO.TO@nano-C anodes, and LMPO and LMPO@nano-C cathodes are investigated by FTIR (Fourier transform infrared spectroscopy produced by Perkin-Elmer, Inc., USA).

**
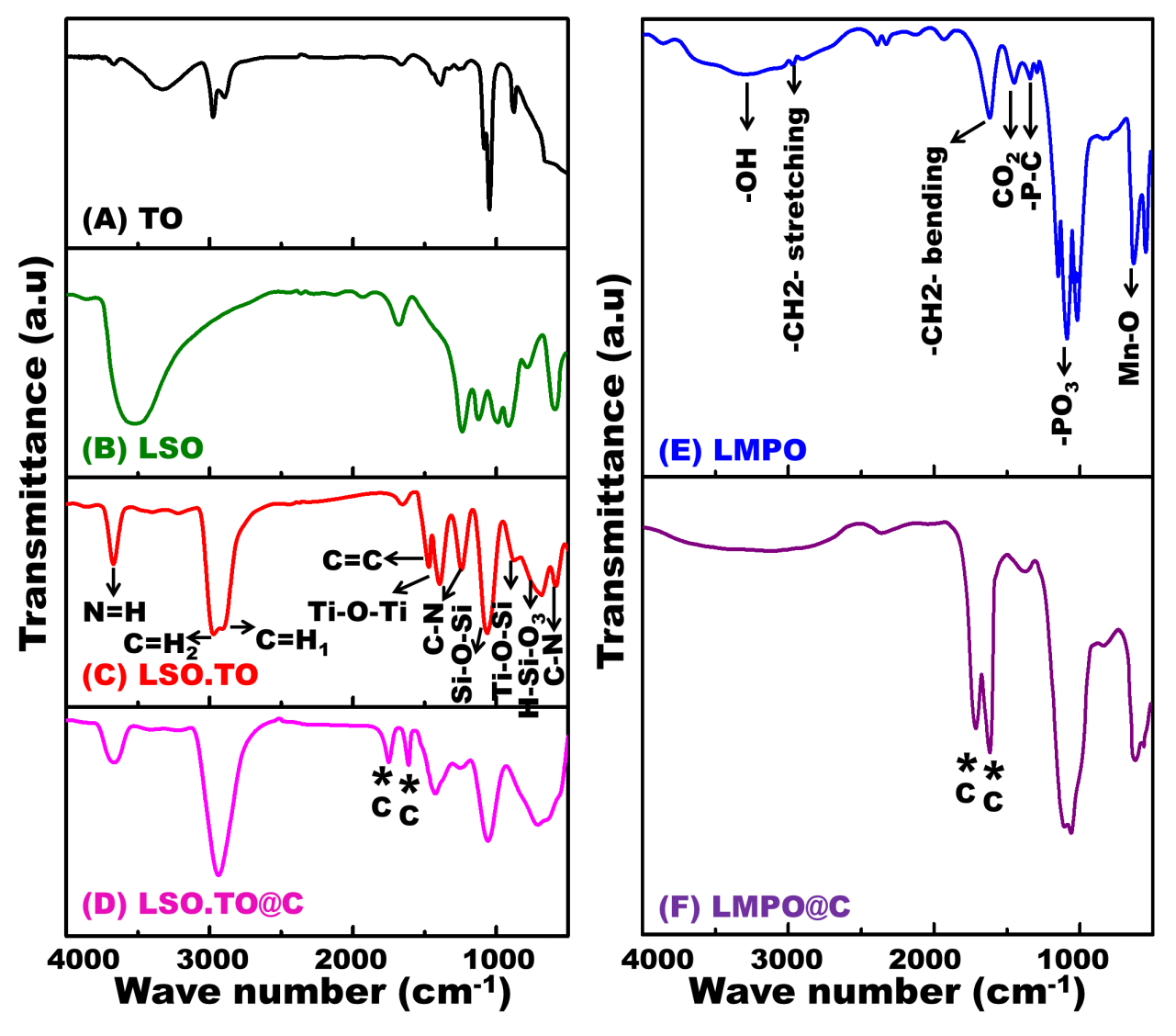
**

**Fig. S7** FT-IR analyses of (A) TO, (B) LSO, (C) LSO.TO, (D) LSO.TO@nano-C, (E) LMPO and (F) LMPO@nano-C architects.

To investigate the formation of anisotropic heterogeneous composites, Fourier transform infrared (FT-IR) analyses are performed for anodic TiO_2_, Li_2_SiO_3_, Li_2_SiO_3_.TiO_2_ and [Li_2_SiO_3_.TiO_2_@nano-C](mailto:Li2SiO3.TiO2@C), and cathodic LiMnPO_4,_ LiMnPO_4_@nano-C compositions, as shown in supporting information, Fig. S7. The LSO.TO shows the presence of C–O at 1246 cm^-1^ and strong bands 2895 cm^-1^ and 2980 cm^-1^ owing to C=H_1_ and C=H_2_ vibration, Fig. S7C. Strong bands of C=C observed at 1484, Ti-O-Ti at 1400 cm^-1^, Si-O-Si at 1066 cm^-1^, H-Si-O_3_ at 714 cm^-1^ and N–H at 3369 cm^-1^. Moreover, formation of Si-O-Ti framework is so limited and described by weak peak at 872 cm^-1^. Therefore, FT-IR results provide evidence of the consistence of Ti-O-Ti and Si-O-Si frameworks with much less formation of Si-O-Ti system. This finding indicates the formation of pure Li_2_SiO_3_ (LSO) rod-pole core that covered by anatase TO shell-wiry needles. FT-IR spectrum of LMPO shows peaks at 540, 1090, 1440, 3250 cm^−1^, attributing the formation of Mn-O, -CPO_3_, P-C and -CH_2_ stretching vibrations, respectively, Fig. S7(E). On other hand, the existed peaks at 1536, 1622 and 2388cm^−1^ may be due to the bending vibration of -CH2-, surface adsorbed water molecules, and the presence of environmental CO_2_ molecules during the synthesis conditions of LMPO, respectively. Also, the wide band at 3292 cm^−1^ indicates the adsorbed water molecule by LMPO sample. Therefore, FT-IR result ensures the present of MnP framework, although it is consisted of amorphous pore walls. The C-coated anode/cathode samples exhibit characteristic peaks (Fig.S7 (D and F), star marks), which indicate the formation organic-inorganic (or carbon-oxygen bonding) contents, as shown with FTIR spectra of LSO.TO@nano-C at 1606 and 1755 cm^-1^. FTIR spectra of LMPO@nano-C at 1615 and 1718 cm^-1^ suggests the existence of C=O stretching.

**S8. Surface and chemical binding structure of anisotropic architectures**

Raman spectroscopy is a powerful tool enabling to give a real evidence of the effective synthesis control based on heterogeneous-particle diffusion methodology for formulation of LMPO/C and LSO.TO@nano-C composites. In addition, Raman spectra usually shed lights of the successful fabrication of anisotropic surface heterogeneity of multi-reactive components aligned along LMPO/C and LSO.TO@nano-C super-hierarchy formulated cathode/anode architects.


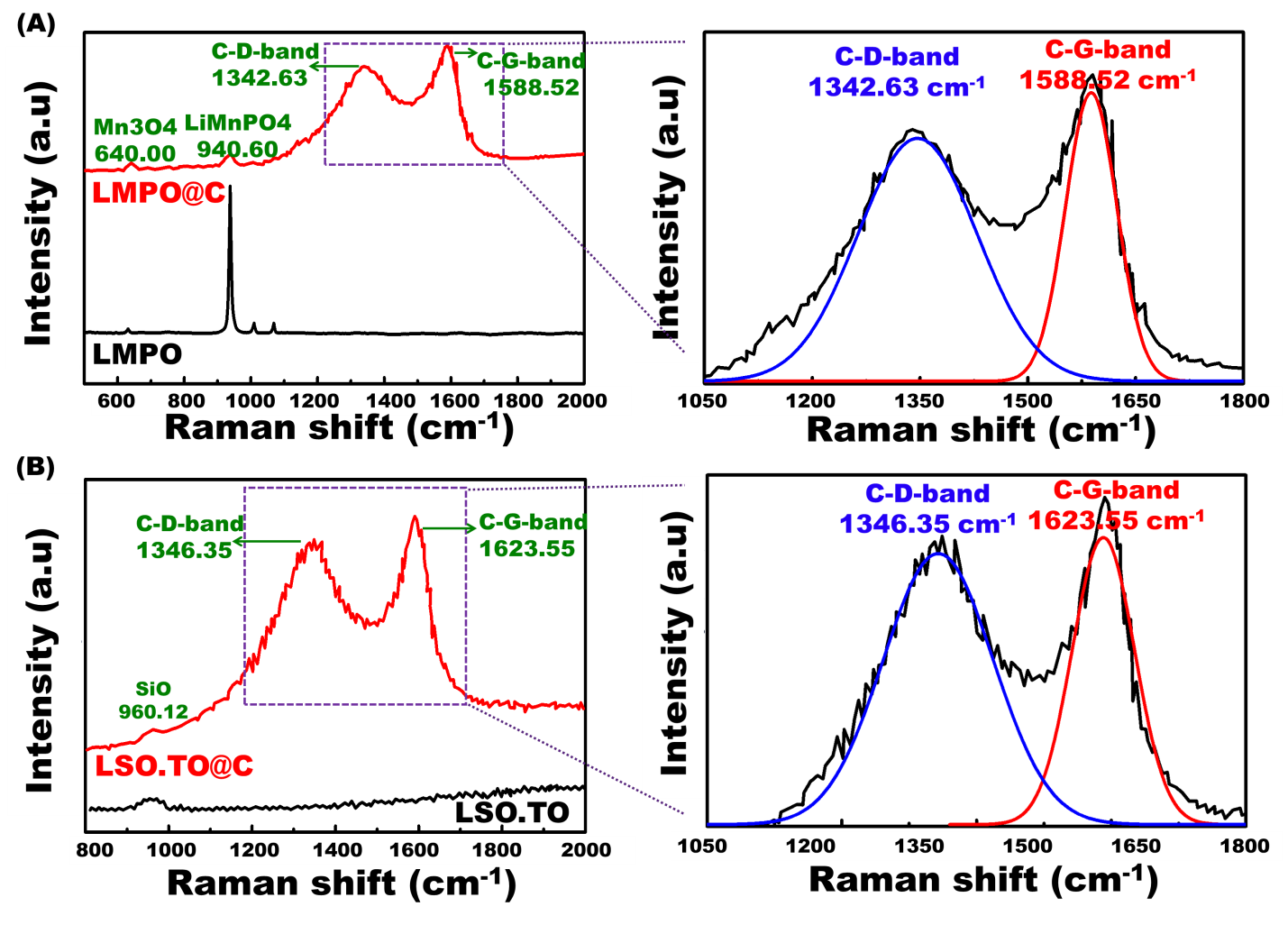


**Fig. S8.** **(A, B)** Raman spectra of LMPO@nano-C and LSO.TO@nano-C composites, respectively

Raman spectroscopy results for LMPO/C and LSO.TO@nano-C composites are shown in Fig.S8. Fig.S8 (A) and its enlarged data (inset of A) show Raman spectra for LMPO@nano-C cathode. The bands at 640.0 and 940.6 cm^−1^ are corresponded to symmetric mode of Mn_3_O_4_ and PO_4_^3−^groups in LMPO@nano-C, referring to the C-coating shell-thin. Two peaks at 1342.63 and 1588.52 cm^−1^ are attributed to D and G bands of nano-carbon bumps in LMPO@nano-C, respectively. The C-D-peak indicates to a disordered carbon of highly defective graphite. The C-G- peak is related to (graphite, in-plane vibrations with E_2g_ symmetry). Fig.S8. (B) and its enlarged data (inset of B) show the Raman spectra for LSO.TO/C composite with peak values at 960.12 cm^−1^, specifying to intra-molecular stretching methods of SiO in LSO.TO architect sample. The existence of C-D-band is due to the disorder-peak related to extremely defective graphite at 1346.35 cm^−1^. The C-G-band at 1623.55 cm^−1^ is attributed to the vibrations with *E2g* mode symmetry of graphite for LSO.TO/C composites. Together, according to Raman and FTIR analyses, a thin nano-carbon-layer could be decorated or wrapped along the outer surface of LSO.TO@nano-C anode/ LMPO@nano-C cathode architectures, which are partially cross-linked via C=C , C=N, C=O bonds after carbonization.

**S9. Surface binding and chemical composition**

The XPS (X-ray photoelectron spectroscopy) is applied to investigate the homogenous composition formation, surface binding energy, and thermodynamically binding composites of LSO.TO@nano-C anode with stable LSO- rod-core @ TO-feathery@ C-shell structure, and LMPO@nano-C layer-by-layer hierarchy cathode via heterogeneous-particle diffusion synthesis protocol (Figure S9 and Table S1).

Table S1 shows the de-convoluted XPS data of LSO.TO@nano-C and LMPO@nano-C composites and their components. The atomic concentrations are calculated by using the survey spectra as following Table S1:

***Table S1. The atomic concentrations (atomic %) for Li 1s, C 1s, O 1s, Si 2p, Ti 2p, Mn 2p, P 2p and F 1s of LSO.TO@nano-C and LMPO@nano-C composites.***

|  | Atomic Concentration (Atomic %) | | | | | | | |
| --- | --- | --- | --- | --- | --- | --- | --- | --- |
| Sample | Li 1s | C 1s | O 1s | Si 2p | Ti 2p | Mn 2p | P 2p | F 1s |
| LSO.TO@nano-C | 3.4 | 5.4 | 63.5 | 12.0 | 15.6 | - | - | 0.1 |
| LMPO@nano-C | 1.8 | 8.1 | 61.1 | - | - | 14.8 | 14.2 | - |

**
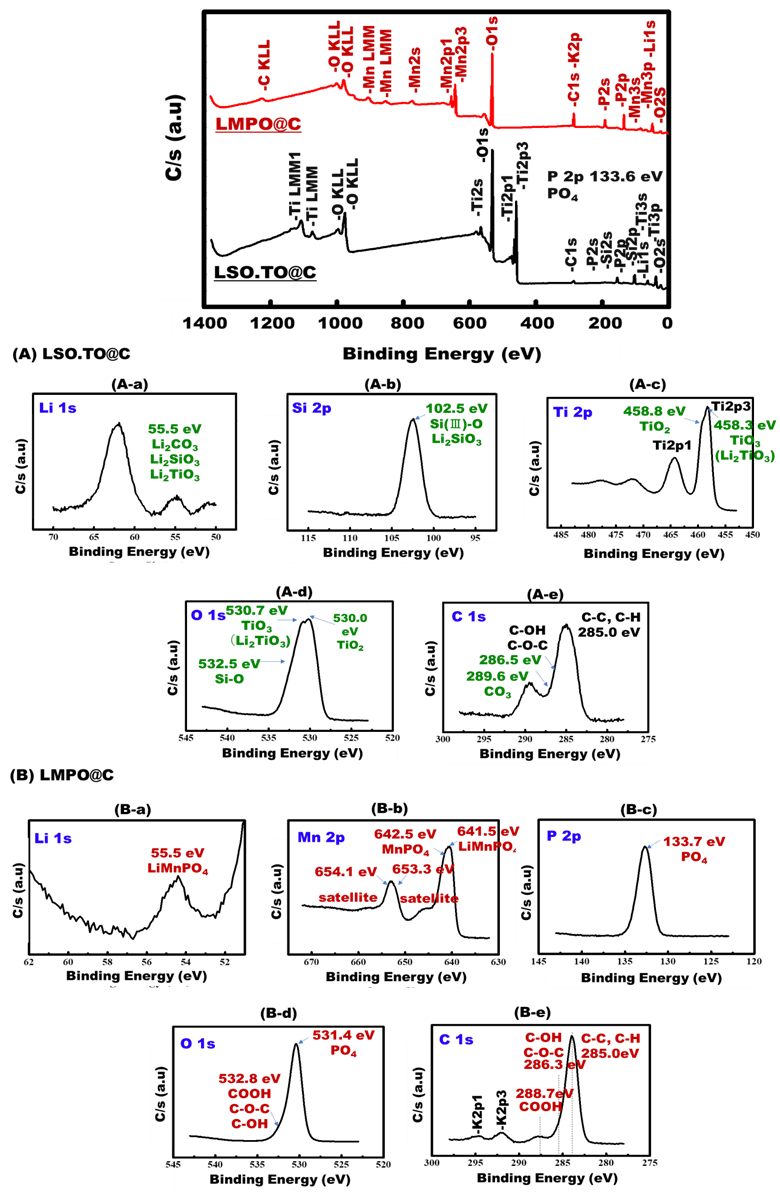
**

**Fig. S9** XPS survey spectrum of the prepared **(A)** LSO.TO@nano-C: **(A-a)** spectra for Li 1s, **(A-b)** spectra for Si 2p, **(A-c)** spectra for Ti 2p, **(A-d)** spectra for O 1s, **(A-e)** spectra for C 1s for LSO.TO@nano-C composites, and **(B)** LMPO@nano-C: **(B-a)** spectra for Li 1s, **(B-b)** spectra for Mn 2p, **(B-c)** spectra for P 2p, **(B-d)** spectra for O 1s, **(B-e)** spectra for C 1s for LMPO@nano-C composites**,** respectively.

The XPS spectra show a real evidence of the compositions and valences of the architects formed from the atomic-scale framework of LSO.TO@nano-C and LMPO@nano-C composites. XPS finding indicates the oxidation states of LSO.TO@nano-C composite anode, Fig. S9(A). The characteristic peaks at 102.5 eV are assigned the specific Si 2p binding energy. The binding energy features of Ti 2p and C 1s are 458.8eV and 286.5eV respectively. The well-defined spectra of the Ti 2p and C 1s peaks indicate the formation of TiO_2-x_-C composite that specifically assigned to TiO_2_@C architects. As shown in Fig. S9(B), the Mn2p binding energy (642.5 eV) of LMPO@nano-C indicates the Mn^3+^ oxidation state in the LMPO. The P2p binding energy (133.7 eV) indicates the P^5+^ oxidation-state valences in the LMPO. The O1s peak at 532.8 eV indicates the valence of O^2-^ in MnPO_4_.

Together, XPS result indicates that the hexagon MnPO_4_ architect is successfully synthesized, in agreement with XRD profiles. The Cls BE to 285eV binding energy (BE) is exhibited as shown in Fig. S9(A). The Mn2p and O1s BE peaks at 711.2 and 531.5 eV indicate the formation of Mn^2+^ and O^2-^ valence states. The 133.7 eV peak of P2p indicates the formation of P^+5^ valences. The peak at 55.5 eV indicates the lithium binding energy. Together, the XRD and XPS data indicate the formation of LSO.TO and LMPO crystal structure with chemical composition domains of Li_2_SiO_3_.TiO_2_@nano-C and LiMnPO_4_@nano-C; respectively.

**S10.** **Cyclic voltammetry measurements (CV) of TO and LSO anodes**


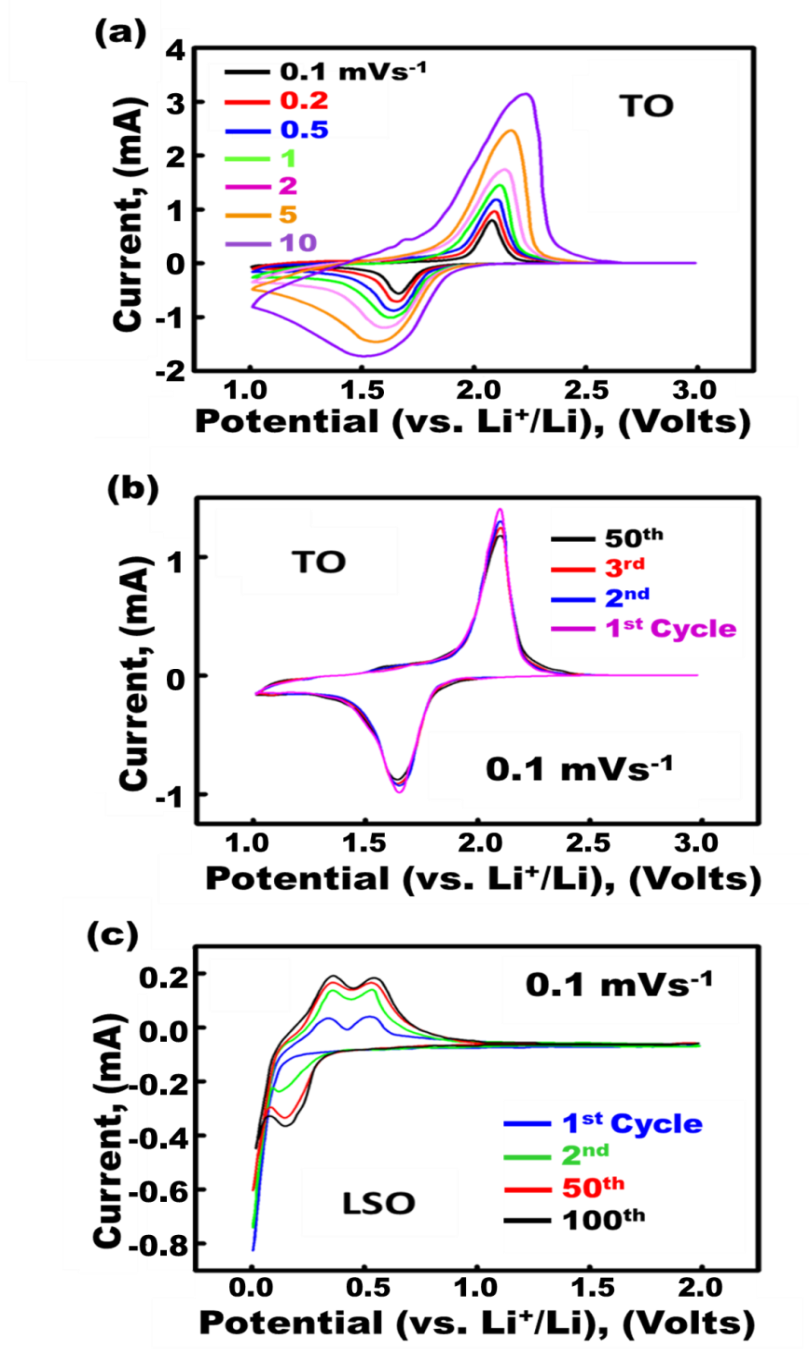


**Fig. S10** Cyclic voltammograms shows a comprehensive analysis of the electrochemical performance of the anode half-cell CR2032 coin-type tests with a Li counter electrode). **(a)** cotton shape anatase TO at different sweep rate (0.1,0.2,0.5,1,5,10 and 20 mVs^-1^), (**b)** TO at different cycle numbers from 1-50 cycles at 0.1 mVs^-1^, **(c)** LSO nanorods at different cycle numbers from 1-50 cycles at 0.1 mVs^-1^. All electrochemical measurements for half-cell TO and LSO anodes are operated within voltage range of (1.0-3.0 V) and (0.005-2.0 V) respectively, at room temperature.

Figure S10(a) shows typical cyclic voltammogram curves for anatase phase TO-cotton-shape over the voltage window of 1–3 V vs. Li/Li^+^ at several scan rates (0.1, 0.2, 0.5, 1, 2, 5 and 10 mV/s). The oxidation/reduction peaks are observed at 2.07/1.67 V. Cyclic voltammograms of anatase-TO within 1^st^, 2^nd^, 3^rd^ and 50^th^ cycles that are investigated at the rate 1mVs^-1^ within a potential range from 1.0 to 3.0 V, as shown in Fig. S10 (b). Two peaks are observed at 1.64 V and 2.1 V related to (i) cathodic reduction process of Ti^4+^ to Ti^3+^ states and (ii) the anodic oxidation of Ti^3+^ to Ti^4+^ sweeps, respectively. The reduction/oxidation process is attributed to the Li^+^-insertion (lithiation) and Li^+^-extraction (delithiation) of Li_x_TiO_2_, respectively. The overlapped CV curves refer to excellent cycling of TO and good reversibility.

CV curves of LSO nanorods at 1^st^, 2^nd^, 50^th^ and 100^th^ cycles are shown in Fig.S10 (c) within a potential region from 0.005V to 2 V at 0.1mVs^-1^. It is evident that 1^st^ CV curve is different from that subsequent curves. It is observed that there are no anodic peaks at first cycle. Once the peak appeared then gradually increased for next cycles, which may be attributed to the activation process of silicon composites. After first cycle, CV curve almost suggests that the Si-based reduction and oxidation peaks are attributed to lithiation and delithiation processes of LSO; respectively. The oxidation peak around 0.55 V may correspond to growth of solid electrolyte interface (SEI) layer onto surface of LSO anodic composite. The reduction peak at 0.15 V indicates the LSO-phase transition.

**S11.** **Electrochemical measurements of TO and LSO anodes**


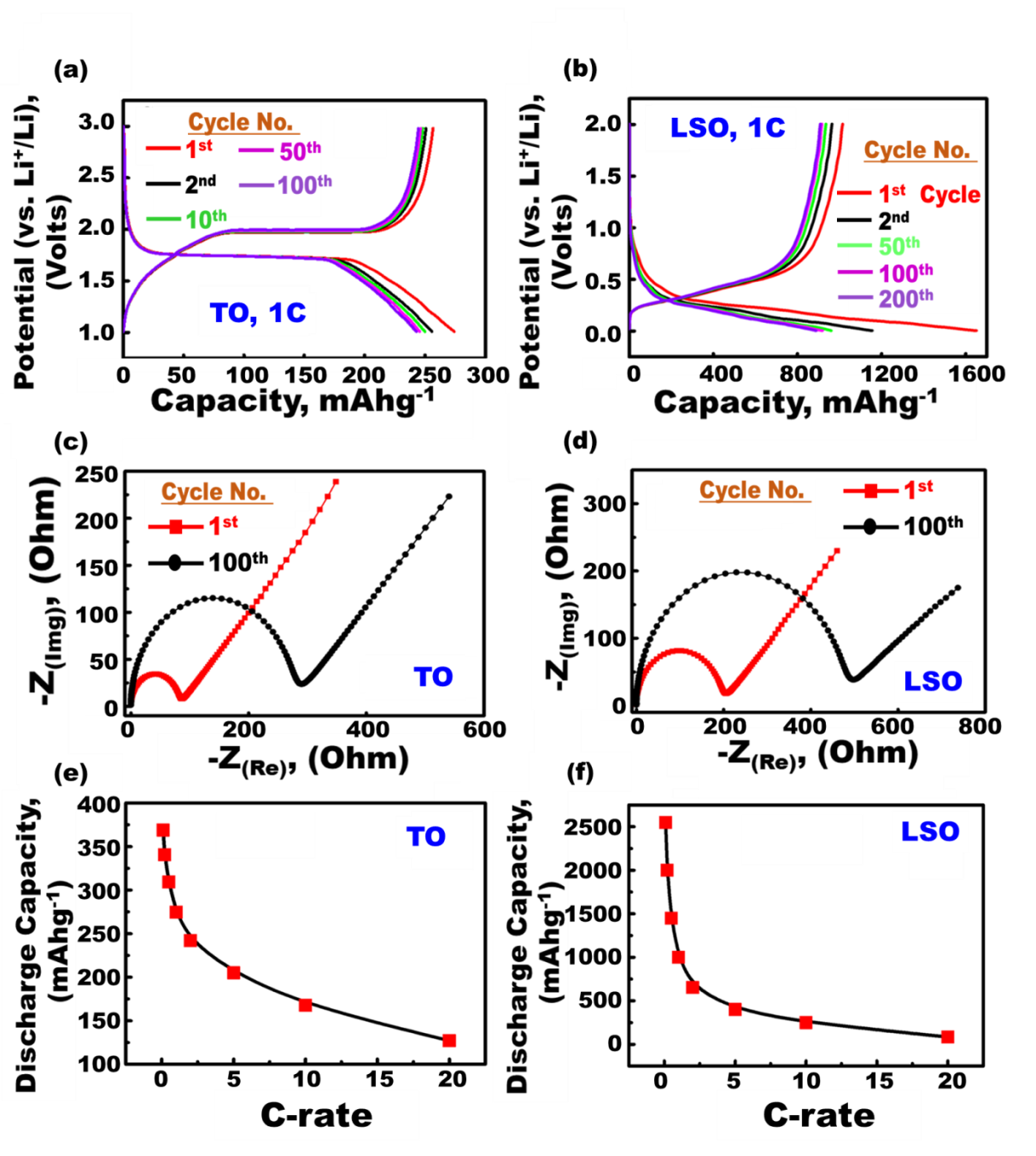


**Fig. S11** The charge-discharge voltage profiles at a current rate 1C of half-cell anode in LIBs; (a) TO-anode at different cycles up to 100 cycles, and (b) LSO-anode at different cycles up to 200 cycles. Electrochemical impedance spectroscopy (EIS) results for (c) TO and (d) LSO half-cells anodic electrodes. Behavior of specific discharge capacity in mAhg^-1^ versus current C-rates from 0.1C to 20C for half-cell anodes based (e) TO and (f) LSO. All electrochemical measurements for half-cell TO and LSO anodes are operated within voltage range of (1.0-3.0 V) and (0.005-2.0 V) respectively, at room temperature.

Figure S11 (a and b) displays the charge/discharge potential curves at 1C of half-cell TO and LSO anodes. Figure S11 (a) shows the galvanostatic cycling profiles of half-cell anatase TO anode within voltage range 1.0-3.0 V vs. Li/Li^+^. A clearly potential plateaus at 1.73 and 1.96 V are due to the Li^+^-insertion (lithiation/cathodic reduction) and Li^+^-extraction (delithiation/anodic oxidation) processes, respectively. Evidently, the curve of discharge capacity is classified into three featured behaviors. The first band of discharge capacity is due to formation of solid-electrolyte layers. At this stage, insignificant quantity of Li^+^-ions can be embedded into TO cortex surface electrode, leading to the specific and rapid decrease in voltage (i.e., from open circuit voltage value (OCV~3V) to approximately ~1.7 V). Second, the discharge capacity behavior zone can show a long-range of flatten plateau at 1.7 V. The stability in potential indicates lithiation of electrode surface sites to a half of its vacant crystal of TO octahedral anatase structures. The third discharge capacity stage showed a long-range of gradually-represented stage of potential decrease after stable-plateau zone from 1.7 to 1.0 V vs. Li (i.e., a cut-off voltage pattern). The gradual decrease in potential to its cutoff pattern may be attributed to the large amount of lithiation that can cover the surface structure of the architects (i.e., an interfacial storage stage). Furthermore, the charge/discharge cycling paradigms are recorded at 1^st^, 2^nd^, 10^th^, 50^th^ and 100^th^ cycles, and at 1C rate of TO anode, as shown in Fig. S11(a). Moreover, the 1^st^ cycle charge and discharge capacities for TO are found to be 256.7 and 274.3 mAh g^-1^, respectively. However, well-known irreversible capacity ~20 mAh g^-1^ for anatase anodes may be attributed to growth of solid−electrolyte interface (SEI) layer. On other hand charging paradigms also exhibit three regions of (i) an increase in the capacity from 1 to 1.96 V due to monotonic delithiation process, (ii) a continuous Li^+^-extraction process through the plateau regime, and (iii) curved formation due to the solid-solution interface at 3.00 V.

Regarding the performance of LSO anodes, Figure S11(b) displays the charge-discharge potential curves at 1C. The specific capacity of LSO anode is found to be 1651 mAhg^-1^ at 1^st^ discharge cycle, and at C-rate of 1C. This specific value is 4.4 times higher than that of graphite capacity (i.e., theoretically calculated at 372 mA h g^-1^). Our finding indicates the rapid capacity damage after first and second cycles. However, the capacity probably unchanged with gradually little decay for the subsequent cycles (i.e., after 2^nd^ cycle). The discharge capacity after 200 cycles is 888.2 mAh g^-1^ ~ 53.3%, (i.e., capacity retention from its original value of 1651 mAhg^-1^). This finding indicates a remarkable drop of discharge capacity especially from first to second cycle. Electrochemical impedance spectroscopy (EIS) is studied for LSO half-cell anodic electrodes, see Fig. S11 (c) & (d). The behavior of specific discharge capacity at C-rates from 0.1C to 20C for TO and LSO half-cell anodes is studied, as shown in Fig. S11 (e) and Fig. S11 (f).

Together, as shown in Figure 2, the excellent electrochemical performance and good rate capability of built-in anode LSO.TO@nano-C electrode are related to the following aspects:

1. the anisotropic, multiply heterogeneous components oriented along the 3D-super-hierarchically matured LSO.TO-anode;
2. the hierarchal architectures, with vertically aligned feathery needles upper the surfaces of core–pole rod skewers, enabling multi-vacancy space accommodation of electron/Li^+^ ions for a long-period during cycling along distinctive upper-zone surfaces and entirely vertical vascular and horizontal canal alignment; and
3. the multi-orientational components and directions throughout interior/exterior scale rods that may maximize electron gain; as such, electrons diffuse in and out of the exposed intercellular zigzag, helical, latitudinal, and longitudinal directions. The latter diffusional directions may mitigate the random distribution of dense Li^+^ ion movement along the anode to produce an integral and energy-efficient design.

The outstanding building block LSO.TO@nano-C architecture reduces Li^+^ ion diffusion path at surface-exposed and multi-component reactive plane sites of [111]/[101]-[LSO.TO](mailto:LSO.TO)@nano-C- feathery needles. The electronic conductivity of LSO.TO@nano-C is remarkably improved due to the rapid back and forth transports of the charge carriers through highly conductive 3D-network of dressing nano-C-bumps along LSO.TO@nano-C shell. The nano-C-bump offer key functions of that features:

1. The nano-C-bump shell ingredients may create multi-pool grooves for the contiguous surface layer.
2. The nano-C-bumps are definitely protected the LSO.TO anode texture against volume expansion of Si-composites in the central or top-zone crystal surface during lithiation process.
3. The nano-C-layers lead to rapid capacity fading due to the structural crystal pulverization ^49^.

**S12.** **The mass fraction analysis of individual cell components designated pouch LIB models**

The ordered sets of multiple rolls of coin cells and their packed up in a collar fashion of stacked- layers-in LSO.TO@nano-C (anode) and LMPO@nano-C (cathode) pouch LIB models were designated. The mass fraction of cathode as an individual component in a LIB cell is approximately 46%, see Figs. S12 - S13. The working electrodes are prepared by mixing each active LSO.TO@nano-C anode or LMPO@nano-C cathode architects with nano-carbon, and a linker-based polyvinylidene fluoride (PVDF) in a mass composition ratio of 75: 15: 10, respectively. The mass fraction is controlled the determination of the specific energy density, which is practically equal to 237.6 Wh kg^-1^ for the LMPO@nano-C//LSO.TO@nano-C full-scale LIBs, see supporting S13.


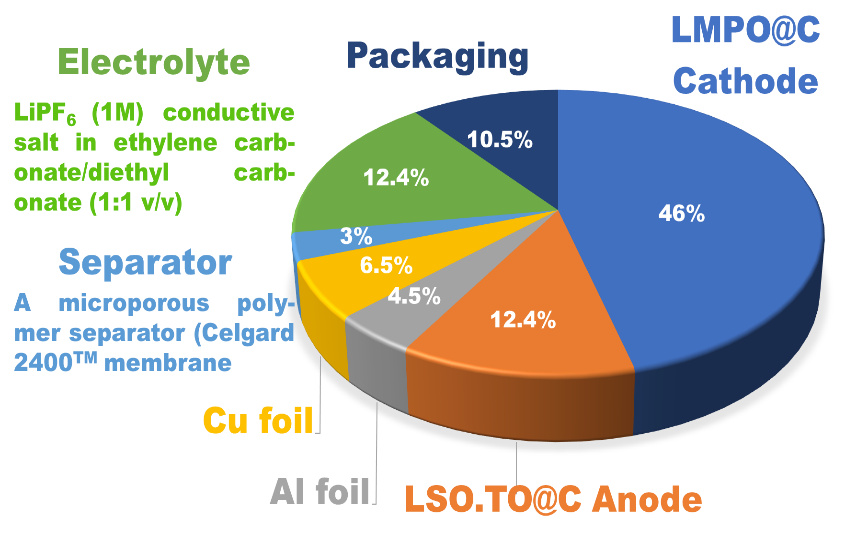


**Fig. S12** Schematic diagram of the pouch cell used for the weight fraction calculation


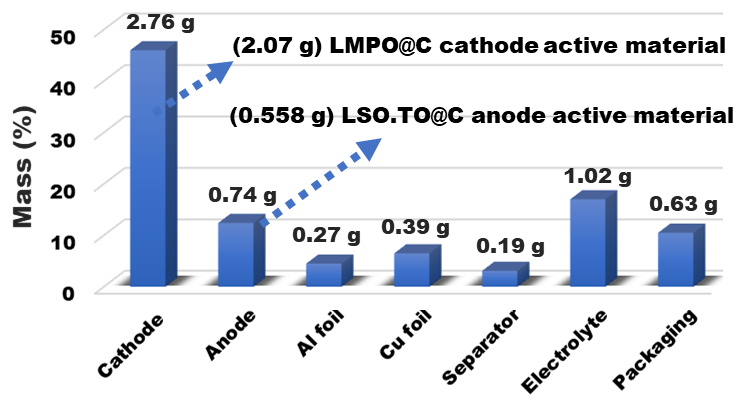


**Fig. S13** Mass fraction of individual components used in the formation of LSO.TO@nano-C//LMPO@nano-C pouch LIB-models

**S13. Determination of specific energy density of (****LSO.TO@nano-C//LMPO@nano-C) full-scale LIB-model**

**S13- A- Theoretical calculation of specific energy density of LMPO@nano-C//LSO.TO@nano-C full-scale LIB:**

In our LMPO@nano-C//LSO.TO@nano-C full-scale LIB design, the nominal open circuit voltage (V_OC_) of positive//negative electrodes would be as follows:

V_OC_ = V_+_ - V_-_

Where V_+_ represents the potential of half-cell of LMPO P-electrode and V_-_ is the potential of LSO.TO@nano-C N-electrode. The nominal voltages known for LMPO and LSO.TO@nano-C cathode and anode are 3.6 V and 0.7 V, respectively. Thus, the full-cell nominal open circuit voltage is as follows:

V_OC_ = 3.6 V – 0.7 V =2.9 V,

The theoretical specific cell capacity of a cell can be calculated in mAhg^−1^ by Faraday’s law:

Q_theoretical_ = (nF) / (3600*M_w_)

Where, F is the Faraday constant, n is the number of charge carrier, and M_w_ is the molecular weight of the architect materials decorated the P- or N- electrode surfaces.

An example is for cathode:

LiMnPO_4_ → MnPO_4_ + 1Li^+^ + 1e^-^

Accordingly, the molecular weight (M_w_) of LiMnPO_4_ is 156.85 ×10^-3^ kg mol^-1^ = 156.85 g mol^-1^, and other factors can be as follows: n=1 and Li^+^=1. F=96 485.3329 sA mol^-1^.

***Therefore, Q value of LiMnPO_4_ (LMPO) electrode can be of 0.17087 Ah g^-1^ = 171 mAh g^-1^.***

According to the abovementioned calculation base, the theoretical specific capacity (Q) for common-used anodic electrode is as follows: nano-sized TiO_2_= 335 mAh/g [Ref-S2- Ref-S4], lithium metasilicate =1965 mAh/g [Ref-S5] and carbon = 372 mAh/g, respectively.

**S13- A- (i) The contribution of each component of LMPO@nano-C and LSO.TO@nano-C full cell specific capacity:**

According to TG and EDS analyses are performed (Figures 1D-I, 1D-II and supporting information Figure S4, S6), we determine the relative composition contents of LiMnPO_4_ and carbon in LiMnPO_4_@nano-C cathode. In addition the compositions of titania, lithium metasilicate and carbon in Li_2_SiO_3_.TiO_2_@nano-C anode is determined. Our finding clearly reveals the presence of Mn, P, O and C elements in LiMnPO_4_@nano-C P-electrode. In addition, the compositions of Si, Ti, O and C elements are formed within [Li_2_SiO_3_.TiO_2_@nano-C](mailto:Li2SiO3.TiO2@C) N-electrode. Carbon content is ~ 4 wt % in the LiMnPO_4_@nano-C compositions.

- ***Therefore, the theoretical specific capacity of cathode P-electrode can be calculated as follows: ~ (372 mAh/g * 0.04 + 171 mAh/g * 0.9996) ~ 185.8 mAh/g.***

In anodic contents, the Ti/Si atomic ratio is approximately 1.9 and carbon content is ~ 4 wt.%, thus the contents of titania and lithium metasilicate can be estimated to be ~ 52 and 44 wt.%, respectively.

- ***Therefore, the theoretical specific capacity of anode N-electrode can be calculated as follows: ~ (******372 mAh/g*** **** 0.04 + 335 mAh/g * 0.52 + 1965 mAh/g * 0.44) ~ 1054 mAh/g.***

**S13- A- (ii) N-, and P-electrode cell capacity balancing**

The cell capacity is controlled by the key value of specific capacity of each of the P- or N-electrode materials presented in the LIB full cell. In fact, the equal capacities for the positive and negative electrodes are presented.

- Assuming an idealized N/P ratio of positive and negative electrode materials in a manner proportionate to their conceptual capacity, then the idealized capacity ratio of LiMnPO_4_@nano-C/ Li_2_SiO_3_.TiO_2_@nano-C is as follows: 185.8 Ah kg^−1^/1054 Ah kg^−1^= 0.178
- Then, the idealized battery with 185.8 Ah of capacity would contain 1 kg of LiMnPO_4_@nano-C and 0.178 kg of Li_2_SiO_3_.TiO_2_@nano-C.
- Because of the combination of mass fraction of both P- and N-electrodes, the utilization of an approximately ~ 41% weight enabled the balance of a practical pouch LIB cell key-component constitutes (i.e., conductive electrolyte, polymeric membrane separator, current electrodes and package cortices, **see Figures S12 and S13).** Therefore, the full scale LIB with a specific capacity of ((185.8 Ah)/(1 kg+0.178 kg))/1.41 is about 111.8 Ah kg^-1^.

The specific energy of the battery would then be obtained by multiplying this resulting capacity and the expected cell voltage (V_OC_) for this combination of P- and N-electrode materials:

***Thus, the specific energy density (i.e., theoretically) of the full-scale battery is = 2.9 V *111.8 Ah kg^-1^ = 324.2 Wh kg^-1^.***

**S13- B- Practical method-based experimental sets of LIBs**

In galvanostatic cycling test (i.e., only at constant current), the specific energy can be calculated graphically from the charge–discharge voltage-capacity profile at 1C as follows:

- The specific energy for the LMPO@nano-C cathode (Wh/kg) can be calculated as follows: (the average working voltage of the sets of LIBs (mid-point voltage) in (V) x maximum discharge capacity delivered by cell (Ahkg^-1^)).
- The average working voltage of the LSO.TO@nano-C//LMPO@nano-C full-scale LIB (mid-point voltage) in (V) is = 3.45 V, and the maximum discharge capacity delivered by cell (Ahkg^-1^) at **1C** = 149.7 Ahkg^-1^.
- ***The specific energy*** ***for the cathode electrode (******Wh/kg) is = 3.45 V x 149.7 Ahkg^-1^ =516.5 Wh/kg.***

Thus, the specific energy density of LIB full-scale (Whkg^-1^) is = the estimated cathodic mass loading in a LIB cell (in pouch-type) x specific energy for the LMPO@nano-C cathode (Whkg^-1^). Based on the mass loading components of well-known pouch LIB cell (see supporting information S12), the cathodic mass-loading component fraction (i.e., LMPO@nano-C cell components) in a full-cell LIB is ~ 46%.

***Therefore, the specific energy density of full-scale LIBs is = 516.5 x 0.46=*** ***237.6 Whkg^-1^.***

The significant value of specific energy density of 237.6 Wh kg^-1^ can improve the EV long-driving range. The outstanding energy density value indicates the key influence of the following:

1. A design configuration of ordered sets of LSO.TO@nano-C (anode) and LMPO@nano-C (cathode) full-cell pouch LIB-types.
2. The multiscale, anisotropic surface heterogeneity of LIB electrodes.
3. The multi-component reactive plane sites, dimension scales and vacancies, and composite textures on their outstanding electrochemical performance.

**S14. The lithium intercalation–deintercalation mechanism and its chemical reactions**

**S14- A- Reactions of Li ions with positive electrode LiMnPO_4_ -** **cathode**

Positive LiMnPO_4_ cathode P-electrode is intercalated compound, in which Li^+^ ions can diffuse-out (discharge) or back-in (charge), as evidenced from Fig. 3 (a). The lithium intercalation/de-intercalation mechanism and its chemical reactions could be summarized as the following:

Cathode - discharged mechanism (lithiation):

- At 3.84 V

MnPO_4_ + xLi^+^ + xe^-^ → LiMnPO_4_

Cathode - charged mechanism (delithiation):

- At 4.23 V

LiMnPO_4_ → MnPO_4_ + xLi^+^ + xe^-^

**S14- B-** **Reactions of Li ions with negative electrode Li_2_SiO_3_.TiO_2_ - anode**

Negative Li_2_SiO_3_.TiO_2_ N-electrode is intercalated compound, in which Li^+^ ions can diffuse out (discharge) or back in (charge), as evidenced from Fig. 2 (a). The lithium intercalation/de-intercalation mechanism and its chemical reactions could be summarized as the following:

Anode - discharged mechanism (lithiation):

- At 1.7 V

TiO_2_ + xLi^+^ + xe^-^ → Li_x_TiO_2_ (x=1)

- At 0.2 V

Li_2_SiO_3_ + Li^+^ + e^-^ → (3/4) Li_4_SiO_4_ + (1/4)Si

- At 0.0 V

Si + (3.75) Li^+^ + (3.75)e^-^ → (1/4) Li_15_Si_4_

Anode - charged mechanism (delithiation):

- At 2.07 V

Li_x_TiO_2_ → TiO_2_ + xLi^+^ + xe^-^ (x=1)

- At 0.51 V

Li_4_SiO_4_ + (1/3)Si → (4/3)Li_2_SiO_3_ + (4/3)Li^+^ + (4/3)e^-^

- At 0.35 V

Li_15_Si_4_ → 4Si + 15Li^+^ + 15e^-^

In these anodic reactions, reversible structure phases such as LiTiO_2_, Li_15_Si_4_ and Li_4_Si_4_ are decomposed continually into stable forms of TiO_2_, Si and Li_2_SiO_3_, respectively.

**S15. Optimization of full cell based (N/P)_Cap_ balancing capacity ratio**

To improve the safety issues of stacking-layer LSO.TO@nano-C (anode) and LMPO@nano-C (cathode) design configurations of pouch LIB-types, an optimization control of full cell based (N/P)Cap balancing capacity ratio is considered in this study. Therefore, a slight increase of the mass loading capacity of [Li_2_SiO_3_.TiO_2_@nano-C](mailto:Li2SiO3.TiO2@C) anode ((N:P)_Cap_ capacity balancing ratio ≈1.1–1.2: 1) is furthermore required [Ref-S6-S8]. Thus, an optimal tradeoff relationship between both for both battery safety and high specific energy storage can be controlled as follows:

1. Safety factors (i.e., which can be achieved by increasing the mass of N-electrode with (N:P)_Cap_ ratio of >1:1), and
2. Maintaining of the high specific energy density (i.e., which can be achieved at equal capacities of N- and P-electrode, (N:P)_Cap_ ratio of 1:1).

In our proposed stacking-layer LSO.TO@nano-C (anode) and LMPO@nano-C (cathode) design configurations of pouch LIB-types, we consider a reasonable control between these two contrast choices of safety issue and maintaining high specific energy. Therefore, we fabricated our scalable full-cell systems of pouch LIB-models under optimized (N:P)_Cap_ ratio of ≈ 1.07 – 1.1 :1.

**S16. Design of LSO.TO@nano-C (anode) and LMPO@nano-C (cathode) configurations in pouch LIB-types**

In this large-scale pouch LIB-types, well-packed and dense LSO.TO@nano-C-anode (5-layers/10-sides)//LMPO@nano-C-cathode (6-layers/10-sides) coin cells are contiguously connected into a series of the built-in stack layer configuration of pouch LIB-types. These conditions may offer a wide range of controlled LIB coin-cell design in pouch modes with ever-decreasing in flexible movements of electron/Li^+^ ion during lithiation and delithiation cycling.

In order to optimize the full-cell LIBs, the electrode area is selected with the following dimensions of (3*5=15 cm^2^) and (3*4.75 = 14.3 cm^2^) for LMPO@nano-C-cathode and LSO.TO@nano-C-anode, respectively. Thus, the total area of the cathode and anode coverage the cell battery can be around ~150 and ~143 cm^2^; respectively. Therefore, the mass values of cathode and anode stacking layers are 13.8 and 3.9 mg/cm^2^, respectively. Our finding indicates that the areal discharge capacity the LMPO@nano-C and LSO.TO@nano-C electrodes is 1.24 and 1.325 Ah/cm^2^, respectively.

**S17. Estimation of volumetric energy density of LIB-designs**

We formulated the built-in full-scale LIB using stacked-layers-in pouch and 18650-cylindrical-shaped design to measure the volumetric energy density of the LSO.TO@nano-C (anode) and LMPO@nano-C (cathode) LIB-modules.

**A- Stacked layers-based pouch LIB-models**

Based on the electrochemical cell design of full-scale LSO.TO@nano-C//LMPO@nano-C anode//cathode pouch LIB-model, we estimate the volumetric energy densities as follows:

- Volumetric energy density = gravimetric cell energy (Wh/kg)* total cell mass (kg)/ cell active area volume (L)
- Gravimetric cell energy = 237.6 Wh/kg (practically)
- Total cell mass = 6 g = 0.006 kg
- Active area volume (ignoring the margin of the packaging) = 35 mm (width)* 55 mm (length)* 2.7 mm cell thickness = 5197 mm^3^ = 0.005197 L

***Thus, the volumetric energy density of pouch LIB-models=237.6 * 0.006 / 0.005197 = 274.3 Wh/L***

**B- 18650 cylindrical LIB-models**

With respect to our proposed LSO.TO@nano-C//LMPO@nano-C full cell as 18650 cylindrical cells, we designated the full cell with the mass fraction of cathode material of ~40%. We also control design of whole cell mass of ~ 0.039 kg, and cell volume ~ 0.016 L.

Accordingly, the stored energy density = 237.6 Wh/kg * 0.039 kg= 9.26 Wh.

***Therefore, the volumetric energy density of 18650 cylindrical cell = 9.26/0.016 ~ 579.2 Wh/L.***

This volumetric energy density value (i.e., 579.2 Wh/L) is reasonably value. The finding indicates that our design configuration of LSO.TO@nano-C (anode)// LMPO@nano-C (cathode) cylindrical-shaped LIBs is consistent with other 18650-cylindrical LIBs market determination such as 18650-LIBs 250–693 [Wh](https://en.wikipedia.org/wiki/Watt_hour)/[L](https://en.wikipedia.org/wiki/Liter) [Ref-S9 - Ref-S11] like Panasonic NCR18650B [Ref-S10] and NCR18650GA [Ref-S11] used as EV-LIB.

**S18. Morphology evolution of anode and cathode in full-cell after cycles**

The key retention of anode or cathode architectural designs in terms of 3D molding anisotropic super-hierarchy, multiscale architectures, and surface heterogeneity enables the continuous, stable, and facile transport pathways of Li^+^ ion during charging/discharging cycles. We study the effect of the multiple electrochemical charge/discharge cycling profiles on the morphological LiMnPO_4_ @nano-C cathode architects. For instance, the LiMnPO_4_ @nano-C cathode materials are collected after long-term 1000 cycles at rate of 1C, and the morphological architects is investigated by using FE-SEM, as shown in Figure S14 A-a and A-b respectively. The elemental mapping analysis images (EDS) of LiMnPO_4_ @nano-C cathode materials are also shown in Figure S14 A-c.


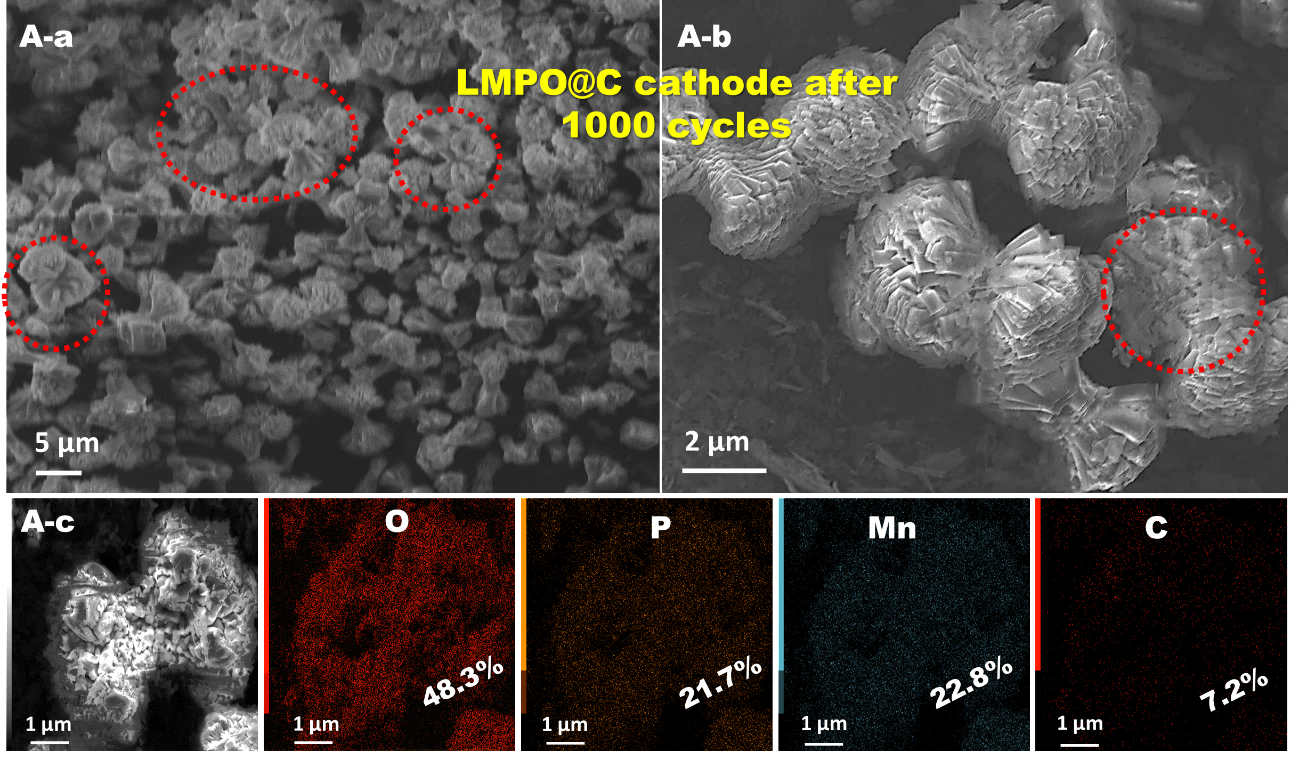


Fig. S14 A(a and b) FE-SEM images of LiMnPO_4_ in full-scale LMPO@nano-C//LSO.TO@nano-C full cell, at different magnifications, after long-term 1000 cycles at 1C. The red dashed lines highlight the regions more affected by the agglomeration of cathode particles. A(c) EDS elemental mapping analyses and images for LiMnPO_4_@nano-C, after long-term 1000 cycles at 1C.

FE-SEM images for cycled LiMnPO_4_@nano-C cathode exhibits the structure stability and uniform orientation in terms of formation of flattened, multi-stacked, dual planar bowtie cantilever layers (antenna-like design) with bi-triangular sheet connected at its cantilever vertex mimic antenna. However, agglomerates and damages in architecture arrangement and morphology is due to multiple cycling up to 1000 cycles at 1C, as shown in Figure S14 A. The structural decomposition/degradation, to some extent, indicates the particular ageing of LMPO@nano-C centering components/sites after 1000 cycles of (LMPO@nano-C//LDO.TO@nano-C) full-scale LIB at current rate 1C comparing to parent LMPO@nano-C electrode, Fig. 1 II(A,B nd C). The finding confirms the excellent structure stability and outstanding electrochemical results even after long-term 1000 cycles, Figure 4 and Scheme 2.

Moreover, EDS elemental mapping after 1000 cycles of LMPO@nano-C cathode in full-scale model (Fig. S14 A-c) confirms that there are no significant changes in the composition dominates of LMPO@nano-C cathode compared with the components and mapping analyses of the parent cathode design (i.e., 1^st^ time used cathode before cycling), Fig. 1-II(D). EDS results (Fig. S14 A-c) show a nearly identical elemental map distribution compared to the pristine electrode 1-II(D) even after 1000 cycles at current rate 1C.

This result conclusively reaffirms that

1. the stability of the LMPO@nano-C//LSO.TO@nano-C full cell LIBs within long-period cycling, and
2. the maintaining of high energy density of cycled full-cell LIBs as a promising candidate for future EVs.

**References:**

[Ref-S1] Park, O. K. *et al.* Who will drive electric vehicles, olivine or spinel? *Energy Environ. Sci.* **4**, 1621 (2011).

[Ref-S2] Chen, J. S., Archer, L. A. & Wen (David) Lou, X. SnO2 hollow structures and TiO2 nanosheets for lithium-ion batteries. *J. Mater. Chem.* **21**, 9912 (2011).

[Ref-S3] Zuniga, L. *et al.* Multichannel hollow structure for improved electrochemical performance of TiO 2 /Carbon composite nanofibers as anodes for lithium ion batteries. *J. Alloys Compd.* **686**, 733–743 (2016).

[Ref-S4] Golubkov, A. W. *et al.* Thermal-runaway experiments on consumer Li-ion batteries with metal-oxide and olivin-type cathodes. *RSC Adv.* **4**, 3633–3642 (2014).

[Ref-S5] Wang, Q., Lu, M. & Miao, J. Li_2_SiO_3_@Li_4_Ti_5_O_12_ nanocomposites as anode material for lithium-ion batteries. *Mater. Technol.* **31**, 471–476 (2016).

[Ref-S6] Zheng, H. *et al.* Correlation between lithium deposition on graphite electrode and the capacity loss for LiFePO_4_ /graphite cells. *Electrochim. Acta* **173**, 323–330 (2015).

[Ref-S7] Kleiner, K., Jakes, P., Scharner, S., Liebau, V. & Ehrenberg, H. Changes of the balancing between anode and cathode due to fatigue in commercial lithium-ion cells. *J. Power Sources* **317**, 25–34 (2016).

[Ref-S8] Andre, D. *et al.* Future generations of cathode materials: an automotive industry perspective. *J. Mater. Chem. A* **3**, 6709–6732 (2015).

[Ref-S9] https://en.wikipedia.org/wiki/Lithium-ion_battery

[Ref-S10] https://www.batteryspace.com/prod-specs/NCR18650B.pdf

[Ref-S11] https://www.orbtronic.com/content/Datasheet-specs-Sanyo-Panasonic-NCR18650GA-3500mah.pdf
